# Hyponatremia in children with respiratory infections: a cross-sectional analysis of a cohort of 3938 patients

**DOI:** 10.1038/s41598-018-34703-1

**Published:** 2018-11-07

**Authors:** Sung Won Park, Son Moon Shin, Moonsun Jeong, Dong-Hee Cho, Keum Hwa Lee, Michael Eisenhut, Andreas Kronbichler, Michael Moritz, Jae Il Shin

**Affiliations:** 1 Department of Pediatrics, Dankook University College of Medicine, Cheil General Hospital & Woman’s Health Care Center, Seoul, Korea; 2 Department of Laboratory Medicine, Dankook University College of Medicine, Cheil General Hospital & Woman’s Health Care Center, Seoul, Korea; 3 Department of Pediatrics, Yonsei University College of Medicine, Seoul, Korea; 4 Luton & Dunstable University Hospital NHS Foundation Trust, Luton, United Kingdom; 5 Medical University Innsbruck, Department of Internal Medicine IV (Nephrology and Hypertension), Innsbruck, Austria; 6 Department of Pediatrics, Children’s Hospital of Pittsburgh of UPMC, The University of Pittsburgh School of Medicine, Pittsburgh, PA USA; 7 Department of Pediatric Nephrology, Institute of Kidney Disease Research, Severance Children’s Hospital, Seoul, South Korea

## Abstract

Hyponatremia can be a life-threatening illness among hospitalized children. The aims of this study were to evaluate the incidence and risk factors of hyponatremia in 3938 children who were admitted to the Cheil General Hospital and Women’s Health Care Center with respiratory infections. Clinical data were collected, and multiplex RT-PCR analyses were done for various microorganisms. Hyponatremia was observed in 531 (13.5%) patients. The incidence of hyponatremia differed according to the respiratory tract infection (*P* < 0.0001) and microorganism (*P* = 0.001). In children with hyponatremia, the age at admission was significantly older (*P* < 0.0001), male gender was more frequent (*P* = 0.019), CRP was higher (*P* < 0.0001), and coinfection with multiple organisms was more common (*P* = 0.001) than in children without hyponatremia. In multivariate analyses, an older age at admission (*P* = 0.006), male gender (*P* = 0.004), and increased CRP (*P* < 0.0001) were independent risk factors. Sodium levels correlated negatively with WBC (*P* = 0.037), CRP (*P* < 0.0001), and number of hospital days (*P* = 0.020). The AUC values of age (0.586, *P* < 0.0001), CRP (0.599, *P* < 0.0001), and blood urea nitrogen (0.559, *P* < 0.0001) were all significant predictors of hyponatremia. This study is the first to show that the incidence of hyponatremia differs according to infecting microorganism and radiological findings.

## Introduction

Hyponatremia is defined as a plasma sodium concentration of less than 135 mEq/L and is one of the most common electrolyte derangements in both adults and children^1^. It can result from either a deficit of sodium or an excess of free water, and the incidence in a small number of cohorts was estimated as 17–45% of hospitalized children^2–5^.

Acute, severe hyponatremia that develops within 48 hours can result in acute cerebral edema and various sequelae, such as headache, lethargy, seizures, and cardiac arrest due to brain stem herniation. Children are more vulnerable than adults to those sequelae because the brain/intracranial volume ratio is higher in children than in adults^6–8^. Recent evidence suggests that even mild chronic hyponatremia can be related to subtle neurologic defects, such as impairments in balance and attention that can increase the incidence of falls^9^.

The most probable cause of hyponatremia is thought to be the non-osmotic release of antidiuretic hormone (ADH) as a result of various clinical conditions, such as fever, hypovolemia, and respiratory tract infections^2,4,10^. Among patients with respiratory tract infections, pneumonia and bronchiolitis are most commonly associated with hyponatremia^3–5^. However, the incidence of hyponatremia according to the etiological involvement of various microorganisms has not yet been studied. As a key event in the pathophysiology of hyponatremia in patients with respiratory tract infections, a syndrome of inappropriate ADH secretion (SIADH) caused by inflammation has been suggested^2,4^, but it has not been evaluated or validated in a sufficiently large number of children. Furthermore, few epidemiologic data are available on hyponatremia in children, and even less is known about hyponatremia in children with respiratory infections. Although hyponatremia is well-known to be associated with respiratory infections in children and adults, that connection has not been studied in a systematic fashion, and no data indicate whether the type of organism, number of organisms, location of infection, or degree of inflammation has any effect on the development of hyponatremia.

Therefore, our aim in this study was to evaluate the incidence of hyponatremia according to the presence of various microorganisms and to elucidate whether the degree of inflammation might be associated with the development of hyponatremia in a large cohort of children hospitalized with respiratory infections.

## Results

The baseline characteristics of the 3938 patients with respiratory tract infections are summarized in Table 1. The mean age of the study population was 22.9 ± 21.7 months (range 7 days–168.0 months), and the sample contained 2195 males (55.7%) and 1743 females (44.3%). The mean duration of hospital stay was 4.4 ± 1.5 days (range 1.0–19.0). The mean age, sex, hospital stay, and C-reactive protein (CRP) levels differed with the radiologic findings and causal microorganisms (Supplementary Tables 1–7). The study population consisted of 177 patients with upper respiratory tract infections (4.5%), 724 patients with acute bronchiolitis (18.4%), 969 patients with acute bronchitis (24.6%), 1978 with interstitial pneumonia (50.2%), and 90 with segmental or lobar pneumonia (2.3%). In multiplex RT-PCR analyses for nasopharyngeal aspirates, human adenovirus (HAdV) was isolated in 137 patients (3.5%), influenza A virus (FLUAV) or influenza B virus (FLUBV) in 107 (2.7%), human parainfluenza virus (HPIV) in 297 (7.5%), human metapneumovirus (HMPV) in 199 (5.1%), human respiratory syncytial virus (HRSV) in 871 (22.1%), human rhinovirus (HRV) in 172 (4.4%), human bocavirus (HBoV) in 576 (14.6%), and no virus was detected in 813 patients (20.6%). *Mycoplasma pneumoniae* infection was detected in 255 patients (6.5%), and coinfection with multiple microorganisms was diagnosed in 511 patients (13.0%).

**Table 1:** Baseline characteristics of the 3938 patients with respiratory infections.

Variables	Total
**Age (months)**	22.9 ± 21.7 (range 7 days–168.0 months)
Sex	
Male	2195 (55.7%)
Female	1743 (44.3%)
**Hospital stay (days)**	4.4 ± 1.5 (range 1.0–19.0)
Diagnosis	
Upper respiratory infection	177 (4.5%)
Acute bronchiolitis	724 (18.4%)
Acute bronchitis	969 (24.6%)
Interstitial pneumonia	1978 (50.2%)
Segmental or lobar pneumonia	90 (2.3%)
Etiologies	
HAdV	137 (3.5%)
FLUAV or FLUBV	107 (2.7%)
HPIV	297 (7.5%)
HMPV	199 (5.1%)
HRSV	871 (22.1%)
HRV	172 (4.4)
HBoV	576 (14.6%)
No virus detected	813 (20.6%)
*Mycoplasma pneumoniae*	255 (6.5%)
Coinfection^†^	511 (13.0%)

HAdV: Human adenovirus, FLUAV/FLUBV: Influenza virus A/B, HPIV: Human parainfluenza virus, HMPV: Human metapneumovirus, HRSV: Human respiratory syncytial virus, HRV: Human rhinovirus, HBoV: Human bocavirus.

^†^Coinfection describes patients who tested positive for more than two microorganisms.

Hyponatremia was observed in 531 (13.5%) of the 3938 patients, among whom 20 had moderate hyponatremia (127–130 mEq/L) (Supplementary Fig. 1). No patient had severe hyponatremia, and two patients had hypernatremia (146 and 151 mEq/L). The incidence of hyponatremia differed according to the radiologic findings (*P* < 0.0001) and microorganisms (*P* = 0.001). The incidence of hyponatremia was highest (44.1%) in children with upper respiratory tract infections. It was 10.5% in acute bronchitis, 13.4% in interstitial pneumonia, 16.7% in segmental and lobar pneumonia, and lowest (9.7%) in acute bronchiolitis. Upper respiratory infections were overrepresented in the hyponatremia group (14.6% vs 2.9%), whereas acute bronchitis and bronchiolitis were underrepresented in the hyponatremia group. In univariate analyses, children with hyponatremia were significantly older (*P* < 0.0001), more likely to be male (*P* = 0.019), and had higher CRP levels (*P* < 0.0001) and coinfections with multiple organisms (*P* = 0.001) than children without hyponatremia. In addition, serum potassium, chloride, total carbon dioxide (tCO_2_), calcium, and phosphorus levels were significantly lower, and serum blood urea nitrogen (BUN) levels were significantly higher in children with hyponatremia than in those without it (Table 2).

**Table 2:** Comparison of demographic data, diagnosis, and laboratory findings of patients with and without hyponatremia.

Variables	Total number of patients (n = 3938)		
	With hyponatremia (n = 531)	Without hyponatremia (n = 3407)	*P* value
**Age (years)**	2.2 ± 1.7	1.9 ± 1.8	<0.0001
**Age (months)**	26.4 ± 20.8	22.3 ± 21.8	<0.0001
**Sex (M/F)**	321/210	1,874/1,533	0.019
**Hospital stay (days)**	4.5 ± 1.5	4.3 ± 1.5	0.093
Diagnosis			
Upper respiratory infection	78 (14.6%)	99(2.9%)	<0.0001
Acute bronchiolitis	70 (13.1%)	654(19.1%)	0.001
Acute bronchitis	102 (19.2%)	867(25.4%)	0.002
Interstitial pneumonia	266 (50.0%)	1712(50.2%)	0.947
Segmental or lobar pneumonia	15 (2.8%)	75(2.2%)	0.371
Laboratory findings at admission			
WBC (/μL)	10,543.3 ± 4,769.6	10,192.0 ± 4,227.5	0.110
CRP (mg/dL)	2.8 ± 3.7	1.8 ± 2.7	<0.0001
Na (mmol/L)	133.2 ± 1.2	137.4 ± 1.7	<0.0001
K (mmol/L)	4.3 ± 0.6	4.4 ± 0.6	0.003
Cl (mmol/L)	100.8 ± 2.5	103.4 ± 2.4	<0.0001
tCO_2_ (mmol/L)	19.7 ± 3.1	21.2 ± 2.8	<0.0001
Ca (mg/dL)	9.2 ± 0.5	9.5 ± 0.5	<0.0001
P (mg/dL)	4.7 ± 1.0	5.1 ± 0.9	<0.0001
ALP (IU/L)	190.2 ± 176.8	191.4 ± 159.0	0.072
AST (IU/L)	46.7 ± 49.0	42.4 ± 31.0	0.050
ALT (IU/L)	24.5 ± 37.5	22.6 ± 33.9	0.258
BUN (mg/dL)	9.6 ± 3.7	8.7 ± 3.4	<0.0001
Creatinine (mg/dL)	0.339 ± 0.082	0.337 ± 0.080	0.618
Laboratory findings at discharge			
Na (mmol/L)	136.9 ± 2.4	138.5 ± 2.1	<0.0001
K (mmol/L)	4.2 ± 0.6	4.5 ± 0.6	<0.0001
Cl (mmol/L)	103.2 ± 2.7	104.0 ± 2.5	0.007
tCO_2_ (mmol/L)	21.6 ± 2.7	22.2 ± 2.5	0.057
**Coinfection**	93 (17.5%)	418 (12.3%)	0.001

WBC: White blood cell, CRP: C-reactive protein, Na: Sodium, K: Potassium, Cl: Chloride, tCO_2_: Total carbon dioxide, Ca: Calcium, P: Phosphorus, ALP: Alkaline phosphatase, AST: Aspartate aminotransferase, ALT: Alanine aminotransferase, BUN: Blood urea nitrogen.

Patients infected by 2 or 3 organisms had a higher incidence of hyponatremia then those with a monoinfection. In the hyponatremia group, the detection of two microorganisms was more common (*P* = 0.004), along with coinfections in general (p = 0.001), than in the without-hyponatremia group. HAdV was the only organism overrepresented in the hyponatremia group (5.3% vs 3.2%). (Table 3).

**Table 3:** Etiology of 3938 patients with and without hyponatremia.

Microorganisms	Total number of patients (n = 3938)		
	With hyponatremia (n = 531)	Without hyponatremia (n = 3407)	*P* value
**No virus detected**	**114 (21.5%)**	**699 (20.5%)**	**0.614**
**One microorganism**	**324 (61.0%)**	**2290 (67.2%)**	**0.005**
HAdV	28 (5.3%)	109 (3.2%)	0.011
FLUAV or FLUBV	13 (2.4%)	94 (2.8%)	0.682
HPIV	41 (7.7%)	256 (7.5%)	0.866
HMPV	29 (5.5%)	170 (5.0%)	0.644
HRSV	108 (20.3%)	763 (22.4%)	0.288
HRV	22 (4.1%)	150 (4.4%)	0.763
HBoV	63 (11.9%)	513 (15.0%)	0.053
Myco	20 (3.8%)	235 (6.9%)	0.011
**Two microorganisms**	**84** (**15.8%)**	**390** (**11.4%)**	**0.004**
HAdV + FLUAV/FLUBV	3 (0.5%)	1 (0.03%)	0.009
HAdV + HPIV	1 (0.2%)	17 (0.5%)	0.497
HAdV + HMPV	1 (0.2%)	4 (0.1%)	0.552
HAdV + HRSV	3 (0.5%)	27 (0.8%)	0.789
HAdV + HRV	9 (1.8%)	23 (0.7%)	0.015
HAdV + Myco	8 (1.5%)	20 (0.6%)	0.019
FLUAV/FLUBV + HPIV	0 (0.0%)	3 (0.1%)	1.000
FLUAV/FLUBV + HMPV	0 (0.0%)	3 (0.1%)	1.000
FLUAV/FLUBV + HRSV	7 (1.3%)	21 (0.6%)	0.073
FLUAV/FLUBV + HRV	3 (0.5%)	2 (0.06%)	0.020
FLUAV/FLUBV + Myco	6 (1.2%)	28 (0.9%)	0.475
HPIV + HMPV	2 (0.4%)	4 (0.1%)	0.188
HPIV + HRSV	0 (0.0%)	10 (0.3%)	0.376
HPIV + HRV	4 (0.8%)	20 (0.6%)	0.555
HPIV + Myco	7 (1.3%)	29 (0.9%)	0.293
HMPV + HRSV	0 (0.0%)	7 (0.2%)	0.604
HMPV + HRV	1 (0.2%)	2 (0.06%)	0.353
HMPV + Myco	8 (1.5%)	21 (0.6%)	0.026
HMPV + HBoV	0 (0.0%)	1 (0.03%)	1.000
HRSV + HRV	3 (0.5%)	42 (1.2%)	0.269
HRSV + Myco	11 (2.1%)	79 (2.3%)	0.723
HRV + Myco	7 (1.3%)	26 (0.7%)	0.192
**Three microorganisms**	**8** (**1.5%)**	**28** (**0.9%)**	**0.123**
HAdV + FLUAV/FLUBV + HRSV	0 (0.0%)	1 (0.03%)	1.000
HAdV + HPIV + HMPV	0 (0.0%)	1 (0.03%)	1.000
HAdV + HPIV + HRV	1 (0.2%)	1 (0.03%)	0.252
HAdV + HPIV + Myco	2 (0.4%)	1 (0.03%)	0.050
HAdV + HPIV + HRSV	1 (0.2%)	0 (0.0%)	0.135
HAdV + HMPV + HRSV	0 (0.0%)	1 (0.03%)	1.000
HAdV + HRSV + HRV	0 (0.0%)	1 (0.03%)	1.000
HAdV + HRSV + Myco	3 (0.5%)	5 (0.2%)	0.081
HAdV + HRV + Myco	0 (0.0%)	2 (0.06%)	1.000
FLUAV/FLUBV + HRSV + HRV	0 (0.0%)	1 (0.03%)	1.000
FLUAV/FLUBV + HRSV + Myco	0 (0.0%)	1 (0.03%)	1.000
FLUAV/FLUBV + HRV + Myco	0 (0.0%)	1 (0.03%)	1.000
HPIV + HRSV + HRV	0 (0.0%)	1 (0.03%)	1.000
HPIV + HRV + Myco	1 (0.2%)	5 (0.2%)	0.581
HMPV + HRSV + Myco	0 (0.0%)	1 (0.03%)	1.000
HMPV + HSV + Myco	0 (0.0%)	1 (0.03%)	1.000
HRSV + HRV + Myco	0(0.0%)	4(0.1%)	1.000
**Four microorganisms**	**1** (**0.2%)**	**0** (**0.0%)**	**0.135**
HAdV + HPIV + HRV + Myco	1 (0.2%)	0 (0.0%)	0.135
**Coinfection total** ^**†**^	**93** (**17.5%)**	**418** (**12.3%)**	**0.001**

HAdV: Human adenovirus, FLUAV/FLUBV: Influenza virus A/B, HPIV: Human parainfluenza virus, HMPV: Human metapneumovirus, HRSV: Human respiratory syncytial virus, HRV: Human rhinovirus, HBoV: Human bocavirus, Myco: *Mycoplasma pneumoniae*.

^†^Coinfection describes patients who tested positive for more than two microorganisms.

In multivariate analyses, an older age at admission (OR 1.007, 95% CI 1.002–1.012, p = 0.006), male gender (OR 1.361, 95% CI 1.105–1.675, p = 0.004), and increased CRP levels (OR 1.093, 95% CI 1.060–1.128, *P* < 0.0001) were independent risk factors for the development of hyponatremia. Coinfection (OR 3.493, 95% CI 0.814–14.998, *P* = 0.092) had borderline significance (Table 4). In a comparison of electrolyte changes among patients (Table 5), serum sodium (*P* < 0.0001), chloride (*P* < 0.0001), and tCO_2_ levels (*P* < 0.0001) were increased in both groups (with and without hyponatremia) at discharge. The mean sodium level in patients with hyponatremia increased from 132.8 mmol/L to 136.9 mmol/L after treatment.

**Table 4:** Multiple logistic regression analyses of laboratory parameters associated with the development of hyponatremia in children with respiratory infections.

Variables	Odds ratio	95% CI	*P* value
Age (months)	1.007	1.002–1.012	0.006
Sex (male)	1.361	1.105–1.675	0.004
CRP	1.093	1.060–1.128	<0.0001
Coinfection^†^	3.493	0.814–14.998	0.092
WBC	1.003	0.979–1.027	0.826
Hospital stay (days)	1.014	0.942–1.091	0.715

CI: Confidence interval, CRP: C-reactive protein.

**Table 5:** Comparison of electrolyte changes between patients with and without hyponatremia.

Group Laboratory findings	Total number of patients		*P* value	Without hyponatremia		*P* value	With hyponatremia		*P* value
	(n = 353)			(n = 531)	(n = 119)		(n = 3407)	(n = 234)	
	At admission Mean ± SD	At discharge Mean ± SD		At admission Mean ± SD	At admission Mean ± SD		At discharge Mean ± SD	At admission	
Na (mmol/L)	135.5 ± 2.4	137.9 ± 2.3	<0.0001	132.8 ± 1.4	136.9 ± 2.4	<0.0001	136.9 ± 1.5	138.5 ± 2.1	<0.0001
K (mmol/L)	4.4 ± 0.6	4.4 ± 0.6	0.732	4.3 ± 0.6	4.2 ± 0.6	0.094	4.4 ±± 0.6	4.5 ± 0.6	0.112
Cl (mmol/L)	102.0 ± 2.5	103.7 ± 2.6	<0.0001	100.1 ± 2.3	103.2 ± 2.7	<0.0001	103.0 ± 2.0	104.0 ± 2.5	<0.0001
tCO_2_ (mmol/L)	20.1 ± 2.9	22.0 ± 2.6	<0.0001	19.1 ± 3.1	21.6 ± 2.7	<0.0001	20.7 ± 2.7	22.2 ± 2.5	<0.0001

SD: Standard deviation, Na: Sodium, K: Potassium, Cl: Chloride, tCO_2_: Total carbon dioxide.

Serum sodium levels correlated positively with serum chloride (r = 0.487, *P* < 0.0001) and tCO_2_ levels (r = 0.231, *P* < 0.0001) and negatively with white blood cell (WBC) counts (r = −0.033, *P* = 0.037) and CRP levels (r = −0.130, *P* < 0.0001). CRP increased the odds ratio of hyponatremia by a small amount (OR 1.093). Also, we found an inverse correlation between serum sodium levels and the length of hospital stay (r = −0.037, *P* = 0.020). However, that correlation differed according to the various microorganisms and radiologic findings (Table 6, Supplementary Table 8).

**Table 6:** Correlations among serum sodium, CRP, and duration of hospitalization.

Valuables	Na and CRP		Na and hospital stay		Na and WBC	
	r	*P* value	r	*P* value	r	*P* value
**Sex**						
Male	(−) 0.147	<0.0001	(−) 0.046	0.030	(−) 0.020	0.355
Female	(−) 0.125	<0.0001	(−) 0.026	0.270	(−) 0.050	0.036
**Total patients**	(−) 0.130	<0.0001	(−) 0.037	0.020	(−) 0.033	0.037
**Diagnosis**						
Acute respiratory infection	(−) 0.146	0.067	(−) 0.069	0.358	(−) 0.080	0.292
Acute bronchiolitis	(−) 0.006	0.884	0.013	0.736	0.030	0.421
Acute bronchitis	(−) 0.106	0.002	(−) 0.026	0.416	0.029	0.372
Interstitial pneumonia	(−) 0.119	<0.0001	(−) 0.068	0.002	(−) 0.041	0.067
Segmental or lobar pneumonia	(−) 0.052	0.643	(−) 0.138	0.194	(−) 0.170	0.110
**Microorganisms**						
No virus	(−) 0.114	0.001	(−) 0.079	0.024	(−) 0.059	0.095
HAdV	(−) 0.086	0.312	(−) 0.044	0.602	(−) 0.044	0.602
FLUAV or FLUBV	0.073	0.456	0.033	0.739	0.059	0.545
HPIV	(−) 0.093	0.108	(−) 0.027	0.642	(−) 0.058	0.334
HMPV	(−) 0.108	0.126	(−) 0.078	0.272	0.000	0.997
HRSV	(−) 0.116	0.001	0.021	0.526	(−) 0.002	0.950
HRV	(−) 0.317	<0.0001	(−) 0.096	0.208	0.156	0.040
HBoV	(−) 0.004	0.982	(−) 0.113	0.007	0.015	0.714
Myco	(−) 0.001	0.991	0.010	0.870	(−) 0.028	0.651
Coinfection^†^	(−) 0.130	<0.0001	(−) 0.037	0.020	(−) 0.033	0.037

Na: Sodium, Hosp: Hospital stay (day), WBC: White blood cell, CRP: C-reactive protein, HAdV: Human adenovirus, FLUAV/FLUBV: Influenza virus A/B, HPIV: Human parainfluenza virus, HMPV: Human metapneumovirus, HRSV: Human respiratory syncytial virus, HRV: Human rhinovirus, HBoV: Human bocavirus, Myco: *Mycoplasma pneumonia*, (−): Negative correlation.

^†^Coinfection describes patients who tested positive for more than two microorganisms.

The area under the curve (AUC) values of age, CRP, and BUN for the prediction of hyponatremia are described in Table 7 and Supplementary Fig. 2. The AUC value of age (0.586, *P* < 0.0001, 95% CI: 0.559–0.613), CRP (0.599, *P* < 0.0001, 95% CI: 0.571–0.628), and BUN (0.559, *P* < 0.0001, 95% CI: 0.531–0.588) were all significant predictors of hyponatremia. The sensitivity, specificity, positive predictive value, negative predictive value, positive likelihood ratio, and negative likelihood ratio of age, CRP, and BUN were determined with different cut-off values. The sensitivity and specificity of age (cut-off value of 18.0 months), CRP (cut-off value of 0.5 mg/dL), and BUN (cut-off value of 9.0 mg/dL) were 57.44% and 55.88%, 60.22% and 52.98%, and 51.32% and 56.05%, respectively (Table 8).

**Table 7:** AUC values of age, CRP, and BUN for the prediction of hyponatremia.

Valuables	AUC	Standard error	^*^P Value	95% CI
Age (months)	0.586	0.014	<0.0001	0.559–0.613
CRP (mg/dL)	0.599	0.014	<0.0001	0.571–0.628
BUN (mg/dL)	0.559	0.014	<0.0001	0.531–0.588

AUC: Area under the curve, CI: Confidence interval, CRP: C-reactive protein, BUN: Blood urea nitrogen.

^*^P values are based on the null hypothesis that AUC = 0.5.

**Table 8:** Diagnostic accuracy of age, CRP, and BUN for the prediction of hyponatremia.

	Sensitivity (95% CI)	Specificity (95% CI)	Positive predictive value	Negative predictive value	Positive LR	Negative LR
**Age (months)**						
>6.0	83.24 (79.8–86.3)	24.77 (23.3–26.3)	10.9	93.0	1.10	0.68
>12.0	76.27 (72.4–79.8)	38.98 (37.3–40.6)	12.2	93.7	1.25	0.61
>18.0	57.44 (53.1–61.7)	55.88 (54.2–57.6)	12.6	92.2	1.30	0.76
**CRP (mg/dL)**						
>0.1	85.16 (81.6–88.3)	22.53 (21.0–24.1)	10.9	93.2	1.10	0.66
>0.4	70.75 (66.4–74.9)	42.08 (40.3–43.9)	12.0	92.8	1.22	0.70
>0.7	60.22 (55.6–64.7)	52.98 (51.2–54.8)	12.5	92.3	1.28	0.75
**BUN (mg/dL)**						
>6.0	82.83 (79.3–85.9)	22.12 (20.7–23.6)	10.6	92.1	1.06	0.78
>7.0	74.72 (70.8–78.4)	32.08 (30.5–33.7)	10.9	91.9	1.10	0.79
>9.0	51.32 (47.0–55.7)	56.05 (54.4–57.7)	11.5	91.2	1.17	0.87

CRP: C-reactive protein, BUN: Blood urea nitrogen, CI: Confidence interval, LR: Likelihood ratio.

## Discussion

Hyponatremia is the most commonly encountered finding in both adults and children with respiratory tract infections. Since Stormont and Waterhouse first reported the association of hyponatremia with pneumonia in 1962^11^, only case reports and a few relevant studies have been published about this relationship in children^3–5,12^.

The incidence of hyponatremia has been reported to be about 30% in a small number of children with pneumonia or acute HRSV bronchiolitis^3–5^, but it has not been estimated in children hospitalized with acute tonsillopharyngitis or acute bronchitis. Moreover, no report has considered the incidence of hyponatremia in children with respiratory tract infections according to the viral etiology, such as adenovirus, metapneumovirus, parainfluenza virus, and rhinovirus. Our study population was the first and largest cohort in the literature evaluating the incidence of hyponatremia in children with respiratory tract infections according to the causal microorganisms or radiologic findings. We found an overall incidence of hyponatremia (<135 mEq/L) of 531 from among 3938 patients (13.5%), and we found no cases with a sodium level of less than 126 mEq/L or seizures. Although a previous small cohort suggested that there was no association between hyponatremia and etiology or radiological patterns in pneumonia^3^, we found that the incidence of hyponatremia was highest (20.7%) in children with adenovirus infection and lowest (7.8%) in those with mycoplasma infection. Although adenovirus was not the most common cause of infection, it was the leading cause of hyponatremia. It may be because the adenovirus infection has a more intenstive systemic inflammatory response as reflected in a greater CRP elevation and more fever than other respiratory viral infections. (Supplementary Table 7).

We also found that the incidence of hyponatremia was highest (44.1%) in children with acute tonsillopharyngitis with no chest X-ray abnormalities, in contrast to the results of the small cohort described by Kaneko *et al*.^12^. They found that deeper inflammatory sites in the respiratory tract were associated with a higher prevalence of hyponatremia in children with respiratory tract infections (pharyngitis 13.3%, bronchitis or bronchiolitis 22.9%, and pneumonia 38.7%). In our study, the WBC and CRP levels in children with acute tonsillopharyngitis were higher than they were in the other groups, and we speculate that the degree of inflammation rather than the inflammatory site might be involved in the development of hyponatremia.

Although the mechanism underlying the development of hyponatremia in respiratory infections is elusive, the traditional concept has been SIADH, in which fever or dehydration reset the osmostat for ADH secretion or increase atrial natriuretic peptide^13^. According to a previous study, SIADH occurs frequently among children hospitalized with pneumonia; Singhi and Dhawan *et al*.^4^ found that 68% of hyponatremia in community acquired pneumonia had characteristics typical of SIADH^4,14^.

There have been studies that hyponatremia is associated with CRP in various diseases^15–17^. Now, we suggest that in patients with respiratory tract infections, SIADH could be caused by the inflammation itself. Recent research revealed that inflammatory cytokines such as interleukin (IL)−1β and IL-6 are involved in the development of hyponatremia associated with inflammatory conditions, and they might be related to ADH secretion^18–20^. Landgraf *et al*.^21^ reported that IL-1β stimulated both the central and peripheral release of vasopressin in rats. In addition, Palin *et al*.^22^ suggested that IL-6 induces activation of arginine vasopressin (AVP) neurons in response to a lipopolysaccharide injection. Most notably, Mastorakos *et al*.^23^ demonstrated that AVP levels were elevated 2 hours after IL-6 injection in six patients. They suggested that IL-6 activated magnocellular AVP-secreting neurons and that it might be involved in the development of inappropriate AVP secretion. Previously, two small studies on pneumonia showed an inverse relationship between inflammatory markers and serum sodium levels^3,24^. In our large cohort, we demonstrated that serum sodium levels were inversely associated with the degree of inflammation (as demonstrated by WBC and CRP levels) in children with various respiratory tract infections, and our logistic regression analysis found that those levels independently predicted the development of hyponatremia. CRP is produced in hepatocytes in response to IL-6.

Very few reports have considered the factors that influence serum sodium levels in children with respiratory tract infections. No one has previously reported the effect of coinfection on the degree of hyponatremia in children with respiratory tract infections; we newly found that coinfection was more frequent in children with hyponatremia than in those without it. Although one study showed no gender difference in serum sodium levels at admission^25^, in our large cohort, males were more prone than females to develop hyponatremia. Wrotek *et al*.^24^ reported that children aged > 4 years with both pneumonia and hyponatremia had higher WBC counts than those without hyponatremia, and we found that the age at admission was significantly higher in children with hyponatremia. Several studies using small cohorts have suggested that the degree of hyponatremia is inversely associated with the length of hospitalization, and we confirmed that finding in our large cohort. Furthermore, we are the first to demonstrate, using multiple logistic regression analyses, that among children hospitalized with various respiratory tract infections, an older age, being male, coinfection, and increased CRP levels are independent risk factors for the development of hyponatremia.

Moderate to severe hyponatremia seems to be substantially more common in developing tropical countries than elsewhere. For example, the 27–31% of Indian children admitted with both community acquired pneumonia and hyponatremia had more severe disease, less favorable outcomes, longer hospitalizations, higher occurrence of complications, and higher mortality rates than those admitted with pneumonia but without hyponatremia^4^. However, we found no adverse effects of hyponatremia on patient outcomes because the patients in our cohort had mild hyponatremia in most cases, and all patients were discharged from the hospital without any complications.

A recent issue in the treatment of hyponatremia has been the tonicity of maintenance intravenous fluids during hospitalization^26–29^. In our study, we used hypotonic intravenous fluids with and without added sodium for hyponatremic and normonatremic patients, respectively. No patient in our cohort experienced aggravation of hyponatremia or seizures, but serum sodium levels stayed the same or were only partially corrected in some patients, suggesting that those patients might have had SIADH, which requires fluid restriction and the use of isotonic fluids. Recent randomized controlled studies and meta analyses have shown that isotonic intravenous fluids reduce the risk of hyponatremia without causing hypernatremia, compared to hypotonic intravenous fluids^27,28^. However, no previous studies have specifically investigated the development of hyponatremia after the use of isotonic vs. hypotonic fluids in children with respiratory tract infections; this subject thus requires further study.

Guppy *et al*. suggested that giving increased fluids to patients with respiratory tract infections, traditionally recommended by doctors, might cause harm because increased ADH secretion could lead to hyponatremia and fluid overload^30^. However, several experts disagreed with that opinion^31,32^, arguing that dehydrated patients with upper respiratory tract infections or bronchitis should be treated by hydration. Furthermore, the two prospective prevalence studies by Guppy *et al*.^4,33^ were on children with moderate to severe pneumonia and no clinical signs of dehydration. Therefore, doctors need to evaluate the clinical status of children with respiratory tract infections, such as oral intake, urine output, and signs of dehydration, before selecting appropriate maintenance intravenous fluids. Nevertheless, because isotonic intravenous fluids are more compatible for correcting dehydration and its related ADH secretion, we recommend the use of isotonic fluids at admission, and the infusion rate can be adjusted according to the status of the patients.

The main limitation of this study is that this study is a retrospective study and therefore it is difficult to precisely assess the severity of the disease and correlation with hyponatremia because it is judged by the investigation of records.

In conclusion, we are the first to evaluate the incidence of hyponatremia according to various microorganisms, along with the factors that affect the development of hyponatremia in a large cohort of children hospitalized with respiratory tract infections. Understanding those characteristics provide insight for clinicians as they choose appropriate maintenance intravenous fluids. Furthermore, our results provide important clues for unraveling the mechanisms of hyponatremia in children hospitalized with respiratory tract infections.

## Methods

### Subjects

We enrolled and retrospectively analyzed data from 3938 children who were admitted to the Cheil General Hospital and Women’s Healthcare Center at the Dankook University College of Medicine for various respiratory tract infections between March, 2011, and February, 2014. This study design and the use of patient information stored in the hospital database were approved by the Institutional Review Board and the research ethics committee of Cheil General Hospital and Women’s Health Care Center. We were given an exemption from the requirement for informed consent because our study was retrospective, personal identifiers were completely removed, and the data were analyzed anonymously. Our study was conducted according to the ethical standards laid down in the 1964 Declaration of Helsinki and its later amendments.

### Laboratory and radiologic investigations

Clinical data such as age, sex, and duration of hospitalization were collected. Laboratory data acquired at admission included complete blood cell counts, the erythrocyte sedimentation rate, CRP, serum electrolytes (sodium, potassium, chloride, and tCO_2_), renal function testing (BUN and creatinine), and liver function testing (aspartate transaminase, alanine transaminase, and alkaline phosphatase). Follow-up electrolyte examinations were carried out in cases with abnormal electrolytes at admission. A chest X-ray was performed in all patients and was interpreted by a pediatric radiologist.

### Definitions

Hyponatremia was defined as a serum sodium concentration (Na^+^) <135 mEq/L. Serum sodium levels were analyzed using a blood test on admission.

The patients were classified into 5 disease categories according to their symptoms and chest X-ray findings.

1. Upper respiratory tract infection: It is defined as an acute infection which involves the upper respiratory tract including the nose, sinuses, pharynx or larynx. We defined upper respiratory infection as diseases including nasal obstruction, sore throat, tonsillitis, pharyngitis, laryngitis, sinusitis, otitis media, and the common cold but the chest X-ray showed normal findings^34^.
2. Acute bronchiolitis: The diagnosis of acute bronchiolitis is typically made by clinical examination^35^. The bronchiolitis is blockage of the small airway in the lungs due to a viral infection It usually only occurs in children less than two years of age^36^. We defined acute bronchiolitis as a disease in which an infant under two years of age develops cough, wheeze, and shortness of breath over one or two days and the chest X-ray showed peribronchial thickening with or without hyperaeration.
3. Acute bronchitis: Bronchitis is inflammation of the bronchi (large and medium-sized airways) in the lungs. Symptoms include coughing up mucus, wheezing, shortness of breath, and chest discomfort^37^. We defined bronchitis as when patients have symptoms of bronchitis and the chest X-ray signs having bronchial wall thickening with increased bronchovascular markings, enlarged vessels without parenchymal lung infection.
4. Interstitial pneumonia: the chest X-ray showed involvement of the areas between the alveoli. Interstitial pneumonia involves the areas in between the alveoli.
5. Segmental or lobar pneumonia: It was defined when the chest X-ray showed the involvement of a single lobe or segment of the lung.

### Multiplex RT-PCR analysis of nasopharyngeal aspirates and the detection of mycoplasma

Strict quality control procedures are used in our hospital. Nasopharyngeal aspirates were collected from patients with respiratory infections using a mucus trap with a sterile suction catheter at admission, and samples were stored at 4 °C on site before testing. Ribonucleic acid (RNA) extraction from clinical samples was performed from the nasopharyngeal aspirates using the Viral Gene-spin TM Viral DNA/RNA Extraction Kit (iNtRON, Seoul, South Korea), and cDNAs were synthesized.

To isolate viral nucleic acids, 200 μl of each respiratory sample was used. Reverse transcription was performed using the Revert Aid TM First Strand cDNA Synthesis Kit (Fermentas, Ontario, Canada). For viral detection, PCR amplification was performed using the Anyplex™ II RV16 Detection Kit (Seegene) with a PTC200 PCR system (MJ Research, Alameda, CA, USA) according to the manufacturer’s instructions.

The kit enabled simultaneous detection of FLUAV, FLUBV, HRSV A and B, HAdV, HMPV, human coronavirus 229 E, human coronavirus NL63, human coronavirus OC43, HPIV −1, −2, −3, −4, HRV A/B/C, and HBoV. Reactions are duplicated in two panels (A and B) for detection of the 16 viruses. PCR was assessed after 15 minutes at 95 °C for transcriptase reverse enzyme inactivation; 50 cycles at 95 °C for 30 seconds, 60 °C for 60 seconds, and 72 °C for 30 seconds; and 1 additional cycle of 55 °C for 30 seconds for completion. The fluorescence was detected with a melting curve step, 55 °C–85 °C (5 seconds/0.5 °C).

*Mycoplasma pneumoniae* infection was detected by Chorus Trio (DIESSE Diagnostica) with a reagent of Chorus *M. pneumoniae* IgM. The reagent is a cartridge that contains 8 compartments. Each compartment contains conjugate, diluted solution, and 3, 3′, 5, 5′ - tetramethyl benzidine. When mounted, 50 μL of serum placed in the first compartment can produce results after 30 minutes. The absorbance is expressed as IgM antibody titer in patient samples, with an index divided by the absorbance value of the correction material. If the index is less than 0.9, the patient is deemed to be negative; if it is 0.9 to 1.1 the patient is deemed ambiguous, and an index of more than 1.1 is positive.

### Statistical analysis

Statistical analysis was performed using SPSS version 20.0 (SPSS, Inc., USA). Continuous variables in two groups were compared using the independent t-test, and if more than 3 groups were compared, we used the analysis of variance. Continuous variables are expressed as the mean ± standard deviation. Categorical variables were analyzed by the chi-square test or Fisher’s exact test. Pearson correlation analysis was performed to examine the relationship between two continuous variables, and multiple logistic regression analyses were performed to find independent predictive factors. In the logistic regression analysis, the CRP was used as a continuous variable, not as normal or abnormal. A *P* value of less than 0.05 was considered statistically significant.

## Electronic supplementary material

Supplementary Dataset 10
