# Supplementary material for: Hyponatremia in children with respiratory infections: a cross-sectional analysis of a cohort of 3938 patients
Source: Sci Rep. 2018 Nov 7;8:16494. doi: 10.1038/s41598-018-34703-1 (PMC6220324; doi:10.1038/s41598-018-34703-1)

**Hyponatremia in children with respiratory infections: a cross-sectional analysis of a cohort of 3938 patients.**

**Sung Won Park^1^, Son Moon Shin ^1^,MoonsunJeong^1^ ,Dong-Hee Cho^2^ ,** **Keum Hwa Lee^3^, Michael Eisenhut^4^ ,**

**Andreas Kronbichler^5^ ,Michael Moritz^6^, Jae Il Shin^3,7^**

1. Department of Pediatrics, Dankook University College of Medicine, Cheil General Hospital & Woman's Health Care Center, Seoul, Korea.
2. Department of Laboratory Medicine, Dankook University College of Medicine, Cheil General Hospital & Woman's Health Care Center, Seoul, Korea
3. Department of Pediatrics, Yonsei University College of Medicine, Severance Children's Hospital, Seoul, Korea
4. Luton &Dunstable University Hospital NHS Foundation Trust, Luton, United Kingdom
5. Medical University Innsbruck, Department of Internal Medicine IV (Nephrology and Hypertension), Innsbruck, Austria
6. Department of Pediatrics, Children's Hospital of Pittsburgh of UPMC, The University of Pittsburgh School of Medicine, Pittsburgh, PA, USA
7. Department of Pediatric Nephrology, Institute of Kidney Disease Research, Severance Children's Hospital, Seoul, South Korea.

**Supplementary Tables**

**Supplementary Table 1. Summary of comparisons of age, duration of hospital stay, CRP,** and sex according to diagnosis and microorganisms

| **Variables** | **Age (months)** | | **Duration of hospital stay (days)** | | **Sex** | |  |
| --- | --- | --- | --- | --- | --- | --- | --- |
|  | **Mean±SD** | ***P* value** | **Mean±SD** | ***P* value** | **Male (n=2195, %)** | ***P* value** |  |
| **Diagnosis** |  |  |  |  |  |  |  |
| Upper respiratory infection | 28.2±20.7 | <0.0001 | 4.2±1.4 | <0.0001 | 94 (53.1) | 0.002 | |
| Acute bronchiolitis | 6.5±7.2 |  | 4.4±1.4 |  | 453 (62.6) |  |  |
| Acute bronchitis | 26.2±24.6 |  | 3.9±1.2 |  | 532 (54.9) |  |  |
| Interstitial pneumonia | 26.3±20.7 |  | 4.5±1.6 |  | 1069 (54.0) |  |  |
| Segmental or lobar pneumonia | 28.4±3.0 |  | 5.0±1.9 |  | 47 (52.2) |  |  |
| **Microorganisms** |  |  |  |  |  |  |  |
| No virus | 24.2±23.4 | <0.0001 | 4.3±1.5 | <0.0001 | 470 (57.8) | 0.001 | |
| HAdV | 31.0±20.1 |  | 4.3±1.3 |  | 81 (57.9) |  |  |
| FLUAV or FLUBV | 30.4±29.4 |  | 4.0±1.2 |  | 59 (55.1) |  |  |
| HPIV | 17.3±14.4 |  | 4.1±1.4 |  | 166 (55.9) |  |  |
| HMPV | 21.7±14.9 |  | 4.2±1.2 |  | 107 (53.2) |  |  |
| HRSV | 12.5±12.8 |  | 4.4±1.5 |  | 488 (56.0) |  |  |
| HRV | 20.4±20.9 |  | 3.9±1.2 |  | 104 (60.5) |  |  |
| HBoV | 21.9±18.3 |  | 4.8±1.7 |  | 348 (60.4) |  |  |
| Myco | 45.6±29.8 |  | 4.4±1.6 |  | 112 (43.9) |  |  |
| Coinfection† | 29.1±22.4 |  | 4.2±1.3 |  | 260 (51.4) |  |  |

HAdV: Human adenovirus, FLUAV/FLUBV: Influenza virus A/B, HPIV: Human parainfluenza virus, HMPV: Human metapneumovirus,

HRSV: Human respiratory syncytial virus, HRV: Human rhinovirus, HBoV: Human bocavirus, Myco: Mycoplasma pneumonia.

†Coinfection describes patients who tested positive for more than two microorganisms.

| **Supplementary Table 2. (1)** **Comparison of age (months) at admission and radiologic findings** | | | | | | | | | | |
| --- | --- | --- | --- | --- | --- | --- | --- | --- | --- | --- |
|  | | Patient  numbers | Mean | Standard  deviation | Standard error | 95% Confidence interval for mean | | Minimum | Maximum | Between- component variance |
|  |  |  |  |  |  | Lower bound | Upper bound |  |  |  |
| Upper respiratory tract infection | | 177 | 28.2142 | 20.73029 | 1.55818 | 25.1391 | 31.2894 | 0.00 | 106.80 |  |
| Acute bronchiolitis | | 724 | 6.5078 | 7.18786 | .26713 | 5.9834 | 7.0323 | 0.00 | 64.80 |  |
| Acute bronchitis | | 969 | 26.1573 | 24.62529 | .79108 | 24.6048 | 27.7097 | 0.00 | 168.00 |  |
| Interstitial pneumonia | | 1978 | 26.3478 | 20.67086 | .46478 | 25.4363 | 27.2593 | 0.00 | 157.20 |  |
| Segmental or lobar pneumonia | | 90 | 33.1973 | 28.44436 | 2.99830 | 27.2398 | 39.1549 | 0.00 | 112.80 |  |
| Total | | 3938 | 22.8938 | 21.73346 | .34633 | 22.2148 | 23.5728 | 0.00 | 168.00 |  |
| Model | Fixed Effects |  |  | 20.27486 | .32309 | 22.2604 | 23.5272 |  |  |  |
|  | Random Effects |  |  |  | 5.74201 | 6.9514 | 38.8362 |  |  | 94.12399 |

**(2) Post-hoc analyses among groups by Bonferroni methods regarding age (months) at admission and** radiologic findings

|  | | | | | | |
| --- | --- | --- | --- | --- | --- | --- |
| Chest x-ray  Group 1 | Chest x-ray  Group 2 | Mean difference between groups  (Groups 1 and 2) | Standard error | Significance | 95% Confidence interval | |
|  |  |  |  |  | Lower bound | Upper bound |
| Upper respiratory infection | Acute bronchiolitis | 21.70639^*^ | 1.70006 | 0.000 | 16.9316 | 26.4812 |
|  | Acute bronchitis | 2.05696 | 1.65730 | 1.000 | -2.5978 | 6.7117 |
|  | Interstitial pneumonia | 1.86641 | 1.59068 | 1.000 | -2.6012 | 6.3340 |
|  | Segmental or lobar pneumonia | -4.98310 | 2.62486 | 0.577 | -12.3553 | 2.3891 |
| Acute bronchiolitis | Upper respiratory infection | -21.70639^*^ | 1.70006 | 0.000 | -26.4812 | -16.9316 |
|  | Acute bronchitis | -19.64943^*^ | 0.99599 | 0.000 | -22.4468 | -16.8521 |
|  | Interstitial pneumonia | -19.83998^*^ | 0.88068 | 0.000 | -22.3135 | -17.3665 |
|  | Segmental or lobar pneumonia | -26.68949^*^ | 2.26610 | 0.000 | -33.0541 | -20.3249 |
| Acute bronchitis | Upper respiratory infection | -2.05696 | 1.65730 | 1.000 | -6.7117 | 2.5978 |
|  | Acute bronchiolitis | 19.64943^*^ | 0.99599 | 0.000 | 16.8521 | 22.4468 |
|  | Interstitial pneumonia | -0.19055 | 0.79501 | 1.000 | -2.4234 | 2.0423 |
|  | Segmental or lobar pneumonia | -7.04006^*^ | 2.23420 | 0.016 | -13.3151 | -0.7650 |
| Interstitial pneumonia | Upper respiratory infection | -1.86641 | 1.59068 | 1.000 | -6.3340 | 2.6012 |
|  | Acute bronchitis | 19.83998^*^ | 0.88068 | 0.000 | 17.3665 | 22.3135 |
|  | Interstitial pneumonia | 0.19055 | 0.79501 | 1.000 | -2.0423 | 2.4234 |
|  | Segmental or lobar pneumonia | -6.84951^*^ | 2.18524 | 0.017 | -12.9870 | -0.7120 |
| Segmental or lobar pneumonia | Upper respiratory infection | 4.98310 | 2.62486 | 0.577 | -2.3891 | 12.3553 |
|  | Acute bronchiolitis | 26.68949^*^ | 2.26610 | 0.000 | 20.3249 | 33.0541 |
|  | Acute bronchitis | 7.04006^*^ | 2.23420 | 0.016 | 0.7650 | 13.3151 |
|  | Interstitial pneumonia | 6.84951^*^ | 2.18524 | 0.017 | 0.7120 | 12.9870 |
| *The mean difference is significant at the 0.05 level. | | | | | | |

| **Supplementary Table 3. (1)** **Comparison of hospital stay (days) and radiologic findings** | | | | | | | | | | |
| --- | --- | --- | --- | --- | --- | --- | --- | --- | --- | --- |
|  | | | | | | | | | | |
|  | | Patient  numbers | Mean | Standard  deviation | Standard error | 95% Confidence interval for mean | | Minimum | Maximum | Between- component variance |
|  |  |  |  |  |  | Lower bound | Upper bound |  |  |  |
| Upper respiratory infection | | 177 | 4.1582 | 1.41737 | 0.10654 | 3.9479 | 4.3684 | 2.00 | 14.00 |  |
| Acute bronchiolitis | | 724 | 4.3785 | 1.36864 | 0.05087 | 4.2786 | 4.4783 | 2.00 | 12.00 |  |
| Acute bronchitis | | 969 | 3.9680 | 1.19053 | 0.03825 | 3.8930 | 4.0431 | 2.00 | 14.00 |  |
| Interstitial pneumonia | | 1978 | 4.5384 | 1.58770 | 0.03570 | 4.4684 | 4.6084 | 1.00 | 19.00 |  |
| Segmental or lobar pneumonia | | 90 | 5.0333 | 1.89292 | 0.19953 | 4.6369 | 5.4298 | 1.00 | 11.00 |  |
| Total | | 3938 | 4.3629 | 1.48162 | 0.02361 | 4.3166 | 4.4092 | 1.00 | 19.00 |  |
| Model | Fixed Effects |  |  | 1.45989 | 0.02326 | 4.3173 | 4.4085 |  |  |  |
|  | Random Effects |  |  |  | 0.18659 | 3.8448 | 4.8809 |  |  | 0.09816 |

**(2) Post-hoc analyses among groups by Bonferroni methods regarding hospital stay (days) and radiologic findings**

| Chest x-ray  Group 1 | Chest x-ray  Group 2 | Mean difference between groups  (Groups 1 and 2) | Standard error | Significance | | 95% Confidence interval | |
| --- | --- | --- | --- | --- | --- | --- | --- |
|  |  |  |  |  |  | Lower bound | Upper bound |
| Upper respiratory infection | Acute bronchiolitis | -0.22026 | 0.12241 | 0.720 | | -0.5641 | 0.1236 |
|  | Acute bronchitis | 0.19018 | 0.11933 | 1.000 | | -0.1450 | 0.5253 |
|  | Interstitial pneumonia | -0.38023^*^ | 0.11454 | 0.009 | | -0.7019 | -0.0585 |
|  | Segmental or lobar pneumonia | -0.87514^*^ | 0.18900 | 0.000 | | -1.4060 | -0.3443 |
| Acute bronchiolitis | Upper respiratory infection | 0.22026 | 0.12241 | 0.720 | | -0.1236 | 0.5641 |
|  | Acute bronchitis | 0.41044^*^ | 0.07172 | 0.000 | | 0.2090 | 0.6119 |
|  | Interstitial pneumonia | -0.15997 | 0.06341 | 0.117 | | -0.3381 | 0.0181 |
|  | Segmental or lobar pneumonia | -0.65488^*^ | 0.16317 | 0.001 | | -1.1132 | -0.1966 |
| Acute bronchitis | Upper respiratory infection | -0.19018 | 0.11933 | 1.000 | | -0.5253 | 0.1450 |
|  | Acute bronchiolitis | -0.41044^*^ | 0.07172 | 0.000 | | -0.6119 | -0.2090 |
|  | Interstitial pneumonia | -0.57041^*^ | 0.05724 | 0.000 | | -0.7312 | -0.4096 |
|  | Segmental or lobar pneumonia | -1.06533^*^ | 0.16087 | 0.000 | | -1.5172 | -0.6135 |
| Interstitial pneumonia | Upper respiratory infection | 0.38023^*^ | 0.11454 | 0.009 | | 0.0585 | 0.7019 |
|  | Acute bronchitis | 0.15997 | 0.06341 | 0.117 | | -0.0181 | 0.3381 |
|  | Interstitial pneumonia | 0.57041^*^ | 0.05724 | 0.000 | | 0.4096 | 0.7312 |
|  | Segmental or lobar pneumonia | -0.49491^*^ | 0.15735 | 0.017 | | -0.9368 | -0.0530 |
| Segmental or lobar pneumonia | Upper respiratory infection | 0.87514^*^ | 0.18900 | 0.000 | | 0.3443 | 1.4060 |
|  | Acute bronchiolitis | 0.65488^*^ | 0.16317 | 0.001 | | 0.1966 | 1.1132 |
|  | Acute bronchitis | 1.06533^*^ | 0.16087 | 0.000 | | 0.6135 | 1.5172 |
|  | Interstitial pneumonia | 0.49491^*^ | 0.15735 | 0.017 | | 0.0530 | 0.9368 |
| *The mean difference is significant at the 0.05 level. | | | | |  | | |

**Supplementary Table 4. (1)** **Comparison of CRP and radiologic findings**

|  | | Patient  numbers | Mean | Standard  deviation | Standard error | 95% Confidence interval for mean | | Minimum | Maximum | Between- component variance |
| --- | --- | --- | --- | --- | --- | --- | --- | --- | --- | --- |
|  |  |  |  |  |  | Lower bound | Upper bound |  |  |  |
| Upper respiratory infection | | 158 | 2.7445 | 3.67221 | 0.29215 | 2.1675 | 3.3215 | 0.00 | 20.50 |  |
| Acute bronchiolitis | | 646 | 0.9075 | 1.79449 | 0.07060 | 0.7689 | 1.0461 | 0.00 | 15.40 |  |
| Acute bronchitis | | 855 | 1.8151 | 2.84053 | 0.09714 | 1.6244 | 2.0057 | 0.00 | 19.00 |  |
| Interstitial pneumonia | | 1641 | 2.2319 | 2.99949 | 0.07404 | 2.0867 | 2.3771 | 0.00 | 21.80 |  |
| Segmental or lobar pneumonia | | 81 | 2.4653 | 3.62546 | 0.40283 | 1.6637 | 3.2670 | 0.00 | 19.30 |  |
| Total | | 3381 | 1.9030 | 2.87064 | 0.04937 | 1.8062 | 1.9998 | 0.00 | 21.80 |  |
| Model | Fixed Effects |  |  | 2.82231 | 0.04854 | 1.8078 | 1.9982 |  |  |  |
|  | Random Effects |  |  |  | 0.37853 | 0.8520 | 2.9540 |  |  | 0.41599 |

CRP: C-reactive protein

**(2) Post-hoc analyses among groups by Bonferroni methods regarding CRP and radiologic findings**

| Chest x-ray  Group 1 | Chest x-ray  Group 2 | Mean difference between groups  (Groups 1 and 2) | Standard error | Significance | | 95% Confidence interval | |
| --- | --- | --- | --- | --- | --- | --- | --- |
|  |  |  |  |  |  | Lower bound | Upper bound |
| Upper respiratory infection | Acute bronchiolitis | 1.83699^*^ | .25049 | .000 | | 1.1334 | 2.5406 |
|  | Acute bronchitis | .92942^*^ | .24440 | .001 | | .2429 | 1.6159 |
|  | Interstitial pneumonia | .51258 | .23509 | .293 | | -.1478 | 1.1729 |
|  | Segmental or lobar pneumonia | .27919 | .38568 | 1.000 | | -.8042 | 1.3625 |
| Acute bronchiolitis | Upper respiratory infection | -1.83699^*^ | .25049 | .000 | | -2.5406 | -1.1334 |
|  | Acute bronchitis | -.90757^*^ | .14713 | .000 | | -1.3208 | -.4943 |
|  | Interstitial pneumonia | -1.32441^*^ | .13109 | .000 | | -1.6926 | -.9562 |
|  | Segmental or lobar pneumonia | -1.55780^*^ | .33267 | .000 | | -2.4922 | -.6234 |
| Acute bronchitis | Upper respiratory infection | -.92942^*^ | .24440 | .001 | | -1.6159 | -.2429 |
|  | Acute bronchiolitis | .90757^*^ | .14713 | .000 | | .4943 | 1.3208 |
|  | Interstitial pneumonia | -.41684^*^ | .11904 | .005 | | -.7512 | -.0825 |
|  | Segmental or lobar pneumonia | -.65023 | .32811 | .476 | | -1.5718 | .2714 |
| Interstitial pneumonia | Upper respiratory infection | -.51258 | .23509 | .293 | | -1.1729 | .1478 |
|  | Acute bronchitis | 1.32441^*^ | .13109 | .000 | | .9562 | 1.6926 |
|  | Interstitial pneumonia | .41684^*^ | .11904 | .005 | | .0825 | .7512 |
|  | Segmental or lobar pneumonia | -.23340 | .32124 | 1.000 | | -1.1357 | .6689 |
| Segmental or lobar pneumonia | Upper respiratory infection | -.27919 | .38568 | 1.000 | | -1.3625 | .8042 |
|  | Acute bronchiolitis | 1.55780^*^ | .33267 | .000 | | .6234 | 2.4922 |
|  | Acute bronchitis | .65023 | .32811 | .476 | | -.2714 | 1.5718 |
|  | Interstitial pneumonia | .23340 | .32124 | 1.000 | | -.6689 | 1.1357 |
| CRP: C-reactive protein * The mean difference is significant at the 0.05 level. | | | | |  | | |

**Supplementary Table 5. (1)** **Comparison of age (months) at admission and microorganisms**

|  | | Patient  numbers | Mean | Standard  deviation | Standard error | 95% Confidence interval for mean | | Minimum | Maximum | Between- component variance |
| --- | --- | --- | --- | --- | --- | --- | --- | --- | --- | --- |
|  |  |  |  |  |  | Lower bound | Upper bound |  |  |  |
| No virus | | 813 | 24.1939 | 23.39421 | 0.82047 | 22.5835 | 25.8044 | 0.00 | 135.60 |  |
| HAdV | | 137 | 31.0126 | 20.05101 | 1.71307 | 27.6248 | 34.4003 | 1.20 | 130.80 |  |
| FLUAV or FLUBV | | 107 | 30.4228 | 29.42718 | 2.84483 | 24.7826 | 36.0630 | 1.20 | 158.40 |  |
| HPIV | | 297 | 17.3390 | 14.38968 | 0.83497 | 15.6958 | 18.9822 | 0.00 | 75.60 |  |
| HMPV | | 199 | 21.5602 | 14.90536 | 1.05661 | 19.4765 | 23.6439 | 1.20 | 73.20 |  |
| HRSV | | 871 | 12.4925 | 12.79870 | 0.43367 | 11.6414 | 13.3437 | 0.00 | 78.00 |  |
| HRV | | 172 | 20.2849 | 20.90632 | 1.59409 | 17.1383 | 23.4315 | 0.00 | 109.20 |  |
| Myco | | 255 | 45.6461 | 29.76662 | 1.86406 | 41.9751 | 49.3171 | 1.20 | 157.20 |  |
| HBoV | | 576 | 21.9465 | 18.28979 | 0.76207 | 20.4497 | 23.4432 | 0.00 | 108.00 |  |
| Coinfection† | | 511 | 29.1410 | 22.35882 | 0.98910 | 27.1978 | 31.0842 | 0.00 | 168.00 |  |
| Total | | 3938 | 22.8938 | 21.73346 | 0.34633 | 22.2148 | 23.5728 | 0.00 | 168.00 |  |
| Model | Fixed Effects |  |  | 20.09246 | 0.32018 | 22.2661 | 23.5215 |  |  |  |
|  | Random Effects |  |  |  | 3.44088 | 15.1100 | 30.6776 |  |  | 80.35594 |

HAdV: Human adenovirus, FLUAV/FLUBV: Influenza virus A/B, HPIV: Human parainfluenza virus, HMPV: Human metapneumovirus, HRSV: Human respiratory

syncytial virus, HRV: Human rhinovirus, Myco: mycoplasma, HBoV: Human bocavirus.

†Coinfection describes patients who tested positive for more than two microorganisms.

**(2) Post-hoc analyses among groups by Bonferroni methods regarding age (months) at admission and microorganisms**

| Virus group 1 | Virus group 2 | Mean difference between groups  (Groups 1 and 2) | Standard error | Significance | 95% Confidence interval | |
| --- | --- | --- | --- | --- | --- | --- |
|  |  |  |  |  | Lower bound | Upper bound |
| No virus | HAdV | -6.81861^*^ | 1.85562 | 0.011 | -12.8738 | -.7634 |
|  | FLUAV or FLUBV | -6.22886 | 2.06628 | 0.117 | -12.9715 | 0.5138 |
|  | HPIV | 6.85496^*^ | 1.36230 | 0.000 | 2.4095 | 11.3004 |
|  | HMPV | 2.63375 | 1.58910 | 1.000 | -2.5518 | 7.8193 |
|  | HRSV | 11.70141^*^ | 0.97983 | 0.000 | 8.5041 | 14.8988 |
|  | HRV | 3.90906 | 1.68633 | 0.922 | -1.5937 | 9.4119 |
|  | Myco | -21.45217^*^ | 1.44213 | 0.000 | -26.1581 | -16.7462 |
|  | HBoV | 2.24749 | 1.09428 | 1.000 | -1.3233 | 5.8183 |
|  | Coinfection† | -4.94703^*^ | 1.13428 | 0.001 | -8.6484 | -1.2457 |
| HAdV | No virus | 6.81861^*^ | 1.85562 | 0.011 | .7634 | 12.8738 |
|  | FLUAV or FLUBV | 0.58975 | 2.59224 | 1.000 | -7.8692 | 9.0487 |
|  | HPIV | 13.67356^*^ | 2.07510 | 0.000 | 6.9021 | 20.4450 |
|  | HMPV | 9.45235^*^ | 2.23057 | 0.001 | 2.1736 | 16.7311 |
|  | HRSV | 18.52002^*^ | 1.84669 | 0.000 | 12.4939 | 24.5461 |
|  | HRV | 10.72767^*^ | 2.30085 | 0.000 | 3.2196 | 18.2358 |
|  | Myco | -14.63356^*^ | 2.12836 | 0.000 | -21.5788 | -7.6883 |
|  | HBoV | 9.06610^*^ | 1.90988 | 0.000 | 2.8338 | 15.2984 |
|  | Coinfection† | 1.87158 | 1.93308 | 1.000 | -4.4364 | 8.1796 |
| FLUAV or FLUBV | No virus | 6.22886 | 2.06628 | 0.117 | -0.5138 | 12.9715 |
|  | HAdV | -0.58975 | 2.59224 | 1.000 | -9.0487 | 7.8692 |
|  | HPIV | 13.08381^*^ | 2.26545 | 0.000 | 5.6912 | 20.4764 |
|  | HMPV | 8.86260^*^ | 2.40866 | 0.011 | 1.0027 | 16.7225 |
|  | HRSV | 17.93027^*^ | 2.05827 | 0.000 | 11.2138 | 24.6468 |
|  | HRV | 10.13792^*^ | 2.47388 | 0.002 | 2.0652 | 18.2107 |
|  | Myco | -15.22331^*^ | 2.31433 | 0.000 | -22.7754 | -7.6712 |
|  | HBoV | 8.47635^*^ | 2.11515 | 0.003 | 1.5742 | 15.3785 |
|  | Coinfection† | 1.28183 | 2.13612 | 1.000 | -5.6887 | 8.2524 |
| HPIV | No virus | -6.85496^*^ | 1.36230 | 0.000 | -11.3004 | -2.4095 |
|  | HAdV | -13.67356^*^ | 2.07510 | 0.000 | -20.4450 | -6.9021 |
|  | FLUAV or FLUBV | -13.08381^*^ | 2.26545 | 0.000 | -20.4764 | -5.6912 |
|  | HMPV | -4.22121 | 1.84064 | 0.985 | -10.2276 | 1.7851 |
|  | HRSV | 4.84645^*^ | 1.35010 | 0.015 | 0.4408 | 9.2521 |
|  | HRV | -2.94589 | 1.92521 | 1.000 | -9.2282 | 3.3364 |
|  | Myco | -28.30713^*^ | 1.71536 | 0.000 | -33.9047 | -22.7096 |
|  | HBoV | -4.60747 | 1.43533 | 0.060 | -9.2912 | 0.0763 |
|  | Coinfection† | -11.80199^*^ | 1.46605 | 0.000 | -16.5860 | -7.0180 |
| HMPV | No virus | -2.63375 | 1.58910 | 1.000 | -7.8193 | 2.5518 |
|  | HAdV | -9.45235^*^ | 2.23057 | 0.001 | -16.7311 | -2.1736 |
|  | FLUAV or FLUBV | -8.86260^*^ | 2.40866 | 0.011 | -16.7225 | -1.0027 |
|  | HPIV | 4.22121 | 1.84064 | 0.985 | -1.7851 | 10.2276 |
|  | HRSV | 9.06766^*^ | 1.57866 | 0.000 | 3.9162 | 14.2191 |
|  | HRV | 1.27532 | 2.09184 | 1.000 | -5.5508 | 8.1014 |
|  | Myco | -24.08592^*^ | 1.90049 | 0.000 | -30.2875 | -17.8843 |
|  | HBoV | -0.38626 | 1.65214 | 1.000 | -5.7775 | 5.0050 |
|  | Coinfection† | -7.58078^*^ | 1.67890 | 0.000 | -13.0593 | -2.1022 |
| HRSV | No virus | -11.70141^*^ | .97983 | 0.000 | -14.8988 | -8.5041 |
|  | HAdV | -18.52002^*^ | 1.84669 | 0.000 | -24.5461 | -12.4939 |
|  | FLUAV or FLUBV | -17.93027^*^ | 2.05827 | 0.000 | -24.6468 | -11.2138 |
|  | HPIV | -4.84645^*^ | 1.35010 | 0.015 | -9.2521 | -0.4408 |
|  | HMPV | -9.06766^*^ | 1.57866 | 0.000 | -14.2191 | -3.9162 |
|  | HRV | -7.79235^*^ | 1.67649 | 0.000 | -13.2631 | -2.3216 |
|  | Myco | -33.15358^*^ | 1.43062 | 0.000 | -37.8219 | -28.4852 |
|  | HBoV | -9.45392^*^ | 1.07906 | 0.000 | -12.9751 | -5.9327 |
|  | Coinfection† | -16.64844^*^ | 1.11961 | 0.000 | -20.3019 | -12.9949 |
| HRV | No virus | -3.90906 | 1.68633 | 0.922 | -9.4119 | 1.5937 |
|  | HAdV | -10.72767^*^ | 2.30085 | 0.000 | -18.2358 | -3.2196 |
|  | FLUAV or FLUBV | -10.13792^*^ | 2.47388 | 0.002 | -18.2107 | -2.0652 |
|  | HPIV | 2.94589 | 1.92521 | 1.000 | -3.3364 | 9.2282 |
|  | HMPV | -1.27532 | 2.09184 | 1.000 | -8.1014 | 5.5508 |
|  | HRSV | 7.79235^*^ | 1.67649 | 0.000 | 2.3216 | 13.2631 |
|  | Myco | -25.36123^*^ | 1.98250 | 0.000 | -31.8305 | -18.8920 |
|  | HBoV | -1.66157 | 1.74586 | 1.000 | -7.3586 | 4.0355 |
|  | Coinfection† | -8.85609^*^ | 1.77121 | 0.000 | -14.6359 | -3.0763 |
| Myco | No virus | 21.45217^*^ | 1.44213 | 0.000 | 16.7462 | 26.1581 |
|  | HAdV | 14.63356^*^ | 2.12836 | 0.000 | 7.6883 | 21.5788 |
|  | FLUAV or FLUBV | 15.22331^*^ | 2.31433 | 0.000 | 7.6712 | 22.7754 |
|  | HPIV | 28.30713^*^ | 1.71536 | 0.000 | 22.7096 | 33.9047 |
|  | HMPV | 24.08592^*^ | 1.90049 | 0.000 | 17.8843 | 30.2875 |
|  | HRSV | 33.15358^*^ | 1.43062 | 0.000 | 28.4852 | 37.8219 |
|  | HRV | 25.36123^*^ | 1.98250 | 0.000 | 18.8920 | 31.8305 |
|  | HBoV | 23.69966^*^ | 1.51131 | 0.000 | 18.7680 | 28.6313 |
|  | Coinfection† | 16.50514^*^ | 1.54052 | 0.000 | 11.4781 | 21.5321 |
| HBoV | HAdV | -2.24749 | 1.09428 | 1.000 | -5.8183 | 1.3233 |
|  | FLUAV or FLUBV | -9.06610^*^ | 1.90988 | 0.000 | -15.2984 | -2.8338 |
|  | HPIV | -8.47635^*^ | 2.11515 | 0.003 | -15.3785 | -1.5742 |
|  | HMPV | 4.60747 | 1.43533 | 0.060 | -0.0763 | 9.2912 |
|  | HRSV | 0.38626 | 1.65214 | 1.000 | -5.0050 | 5.7775 |
|  | HRV | 9.45392^*^ | 1.07906 | 0.000 | 5.9327 | 12.9751 |
|  | Myco | 1.66157 | 1.74586 | 1.000 | -4.0355 | 7.3586 |
|  | HAdV | -23.69966^*^ | 1.51131 | 0.000 | -28.6313 | -18.7680 |
|  | Coinfection† | -7.19452^*^ | 1.22103 | 0.000 | -11.1790 | -3.2101 |
| Coinfection† | No virus | 4.94703^*^ | 1.13428 | 0.001 | 1.2457 | 8.6484 |
|  | HAdV | -1.87158 | 1.93308 | 1.000 | -8.1796 | 4.4364 |
|  | FLUAV or FLUBV | -1.28183 | 2.13612 | 1.000 | -8.2524 | 5.6887 |
|  | HPIV | 11.80199^*^ | 1.46605 | 0.000 | 7.0180 | 16.5860 |
|  | HMPV | 7.58078^*^ | 1.67890 | 0.000 | 2.1022 | 13.0593 |
|  | HRSV | 16.64844^*^ | 1.11961 | 0.000 | 12.9949 | 20.3019 |
|  | HRV | 8.85609^*^ | 1.77121 | 0.000 | 3.0763 | 14.6359 |
|  | Myco | -16.50514^*^ | 1.54052 | 0.000 | -21.5321 | -11.4781 |
|  | HBoV | 7.19452^*^ | 1.22103 | 0.000 | 3.2101 | 11.1790 |

HAdV: Human adenovirus, FLUAV/FLUBV: Influenza virus A/B, HPIV: Human parainfluenza virus, HMPV: Human metapneumovirus, HRSV: Human respiratory syncytial virus, HRV: Human rhinovirus, Myco: Mycoplasma, HBoV: Human bocavirus.

†Coinfection describes patients who tested positive for more than two microorganisms.

*The mean difference is significant at the 0.05 level.

**Supplementary Table 6. (1)** **Comparison of hospital stay (days) and microorganisms**

|  | | Patient  numbers | Mean | Standard  deviation | Standard error | 95% Confidence interval for mean | | Minimum | Maximum | Between- component variance |
| --- | --- | --- | --- | --- | --- | --- | --- | --- | --- | --- |
|  |  |  |  |  |  | Lower bound | Upper bound |  |  |  |
| No virus | | 813 | 4.3050 | 1.47406 | 0.05170 | 4.2036 | 4.4065 | 2.00 | 14.00 |  |
| HAdV | | 137 | 4.3431 | 1.27440 | 0.10888 | 4.1278 | 4.5584 | 2.00 | 8.00 |  |
| FLUAV or FLUBV | | 107 | 3.9533 | 1.23153 | 0.11906 | 3.7172 | 4.1893 | 2.00 | 9.00 |  |
| HPIV | | 297 | 4.0606 | 1.43427 | 0.08322 | 3.8968 | 4.2244 | 2.00 | 19.00 |  |
| HMPV | | 199 | 4.2010 | 1.23493 | 0.08754 | 4.0284 | 4.3736 | 2.00 | 9.00 |  |
| HRSV | | 871 | 4.4386 | 1.47253 | 0.04989 | 4.3406 | 4.5365 | 1.00 | 18.00 |  |
| HRV | | 172 | 3.9186 | 1.17692 | 0.08974 | 3.7415 | 4.0957 | 1.00 | 9.00 |  |
| Myco | | 255 | 4.4275 | 1.64150 | 0.10279 | 4.2250 | 4.6299 | 2.00 | 14.00 |  |
| HBoV | | 576 | 4.8455 | 1.70281 | 0.07095 | 4.7061 | 4.9848 | 2.00 | 15.00 |  |
| Coinfection† | | 511 | 4.2290 | 1.30925 | 0.05792 | 4.1152 | 4.3427 | 2.00 | 10.00 |  |
| Total | | 3938 | 4.3629 | 1.48162 | 0.02361 | 4.3166 | 4.4092 | 1.00 | 19.00 |  |
| Model | Fixed Effects |  |  | 1.46289 | 0.02331 | 4.3172 | 4.4086 |  |  |  |
|  | Random Effects |  |  |  | 0.09987 | 4.1369 | 4.5888 |  |  | 0.06457 |

HAdV: Human adenovirus, FLUAV/FLUBV: Influenza virus A/B, HPIV: Human parainfluenza virus, HMPV: Human metapneumovirus, HRSV: Human respiratory syncytial

virus, HRV: Human rhinovirus, Myco: Mycoplasma, HBoV: Human bocavirus.

†Coinfection describes patients who tested positive for more than two microorganisms.

**(2) Post-hoc analyses among groups by Bonferroni methods regarding hospital stay (days) and microorganisms**

| Virus group 1 | Virus group 2 | Mean difference between groups  (Groups 1 and 2) | Standard error | Significance | 95% Confidence interval | |
| --- | --- | --- | --- | --- | --- | --- |
|  |  |  |  |  | Lower bound | Upper bound |
| No virus | HAdV | -0.03802 | 0.13510 | 1.000 | -0.4789 | 0.4028 |
|  | FLUAV or FLUBV | 0.35177 | 0.15044 | 0.874 | -0.1391 | 0.8427 |
|  | HPIV | 0.24444 | 0.09919 | 0.619 | -0.0792 | 0.5681 |
|  | HMPV | 0.10404 | 0.11570 | 1.000 | -0.2735 | 0.4816 |
|  | HRSV | -0.13353 | 0.07134 | 1.000 | -0.3663 | 0.0993 |
|  | HRV | 0.38644 | 0.12278 | 0.075 | -0.0142 | 0.7871 |
|  | Myco | -0.12241 | 0.10500 | 1.000 | -0.4650 | 0.2202 |
|  | HBoV | -0.54044^*^ | 0.07967 | 0.000 | -0.8004 | -0.2805 |
|  | Coinfection† | 0.07608 | 0.08258 | 1.000 | -0.1934 | 0.3456 |
| HAdV | No virus | 0.03802 | 0.13510 | 1.000 | -0.4028 | 0.4789 |
|  | FLUAV or FLUBV | 0.38979 | 0.18874 | 1.000 | -0.2261 | 1.0057 |
|  | HPIV | 0.28246 | 0.15108 | 1.000 | -0.2106 | 0.7755 |
|  | HMPV | 0.14206 | 0.16240 | 1.000 | -0.3879 | 0.6720 |
|  | HRSV | -0.09551 | 0.13445 | 1.000 | -0.5343 | 0.3432 |
|  | HRV | 0.42446 | 0.16752 | 0.509 | -0.1222 | 0.9711 |
|  | Myco | -0.08439 | 0.15496 | 1.000 | -0.5901 | 0.4213 |
|  | HBoV | -0.50242^*^ | 0.13905 | 0.014 | -0.9562 | -0.0487 |
|  | Coinfection† | 0.11410 | 0.14074 | 1.000 | -0.3452 | 0.5734 |
| FLUAV or FLUBV | No virus | -0.35177 | 0.15044 | 0.874 | -0.8427 | 0.1391 |
|  | HAdV | -0.38979 | 0.18874 | 1.000 | -1.0057 | 0.2261 |
|  | HPIV | -0.10734 | 0.16494 | 1.000 | -0.6456 | 0.4309 |
|  | HMPV | -0.24773 | 0.17537 | 1.000 | -0.8200 | 0.3245 |
|  | HRSV | -0.48531 | 0.14986 | 0.055 | -0.9743 | 0.0037 |
|  | HRV | 0.03467 | 0.18012 | 1.000 | -0.5531 | 0.6224 |
|  | Myco | -0.47418 | 0.16850 | 0.221 | -1.0240 | 0.0757 |
|  | HBoV | -0.89222^*^ | 0.15400 | 0.000 | -1.3947 | -0.3897 |
|  | Coinfection† | -0.27569 | 0.15553 | 1.000 | -0.7832 | 0.2318 |
| HPIV | No virus | -0.24444 | 0.09919 | 0.619 | -0.5681 | 0.0792 |
|  | HAdV | -0.28246 | 0.15108 | 1.000 | -0.7755 | 0.2106 |
|  | FLUAV or FLUBV | 0.10734 | 0.16494 | 1.000 | -0.4309 | 0.6456 |
|  | HMPV | -0.14040 | 0.13401 | 1.000 | -0.5777 | 0.2969 |
|  | HRSV | -0.37797^*^ | 0.09830 | 0.006 | -0.6987 | -0.0572 |
|  | HRV | 0.14200 | 0.14017 | 1.000 | -0.3154 | 0.5994 |
|  | Myco | -0.36684 | 0.12489 | 0.150 | -0.7744 | 0.0407 |
|  | HBoV | -0.78488^*^ | 0.10450 | 0.000 | -1.1259 | -0.4439 |
|  | Coinfection† | -0.16836 | 0.10674 | 1.000 | -0.5167 | 0.1800 |
| HMPV | No virus | -0.10404 | 0.11570 | 1.000 | -0.4816 | 0.2735 |
|  | HAdV | -0.14206 | 0.16240 | 1.000 | -0.6720 | 0.3879 |
|  | FLUAV or FLUBV | 0.24773 | 0.17537 | 1.000 | -0.3245 | 0.8200 |
|  | HPIV | 0.14040 | 0.13401 | 1.000 | -0.2969 | 0.5777 |
|  | HRSV | -0.23757 | 0.11494 | 1.000 | -0.6126 | 0.1375 |
|  | HRV | 0.28240 | 0.15230 | 1.000 | -0.2146 | 0.7794 |
|  | Myco | -0.22645 | 0.13837 | 1.000 | -0.6780 | 0.2251 |
|  | HBoV | -0.64448^*^ | 0.12029 | 0.000 | -1.0370 | -0.2520 |
|  | Coinfection† | -0.02796 | 0.12224 | 1.000 | -0.4268 | 0.3709 |
| HRSV | No virus | 0.13353 | 0.07134 | 1.000 | -0.0993 | 0.3663 |
|  | HAdV | 0.09551 | 0.13445 | 1.000 | -0.3432 | 0.5343 |
|  | FLUAV or FLUBV | 0.48531 | 0.14986 | 0.055 | -0.0037 | 0.9743 |
|  | HPIV | 0.37797^*^ | 0.09830 | 0.006 | 0.0572 | 0.6987 |
|  | HMPV | 0.23757 | 0.11494 | 1.000 | -0.1375 | 0.6126 |
|  | HRV | 0.51997^*^ | 0.12206 | 0.001 | 0.1217 | 0.9183 |
|  | Myco | 0.01113 | 0.10416 | 1.000 | -0.3288 | 0.3510 |
|  | HBoV | -0.40691^*^ | 0.07856 | 0.000 | -0.6633 | -0.1505 |
|  | Coinfection† | 0.20961 | 0.08152 | 0.457 | -0.0564 | 0.4756 |
| HRV | No virus | -0.38644 | 0.12278 | 0.075 | -0.7871 | 0.0142 |
|  | HAdV | -0.42446 | 0.16752 | 0.509 | -0.9711 | 0.1222 |
|  | FLUAV or FLUBV | -0.03467 | 0.18012 | 1.000 | -0.6224 | 0.5531 |
|  | HPIV | -0.14200 | 0.14017 | 1.000 | -0.5994 | 0.3154 |
|  | HMPV | -0.28240 | 0.15230 | 1.000 | -0.7794 | 0.2146 |
|  | HRSV | -0.51997^*^ | 0.12206 | 0.001 | -0.9183 | -0.1217 |
|  | Myco | -0.50885^*^ | 0.14434 | 0.019 | -0.9799 | -0.0378 |
|  | HBoV | -0.92688^*^ | 0.12711 | 0.000 | -1.3417 | -0.5121 |
|  | Coinfection† | -0.31036 | 0.12896 | 0.726 | -0.7312 | 0.1105 |
| Myco | No virus | 0.12241 | 0.10500 | 1.000 | -0.2202 | 0.4650 |
|  | HAdV | 0.08439 | 0.15496 | 1.000 | -0.4213 | 0.5901 |
|  | FLUAV or FLUBV | 0.47418 | 0.16850 | 0.221 | -0.0757 | 1.0240 |
|  | HPIV | 0.36684 | 0.12489 | 0.150 | -0.0407 | 0.7744 |
|  | HMPV | 0.22645 | 0.13837 | 1.000 | -0.2251 | 0.6780 |
|  | HRSV | -0.01113 | 0.10416 | 1.000 | -0.3510 | 0.3288 |
|  | HRV | 0.50885^*^ | 0.14434 | 0.019 | 0.0378 | 0.9799 |
|  | HBoV | -0.41804^*^ | 0.11003 | 0.007 | -0.7771 | -0.0590 |
|  | Coinfection† | 0.19849 | 0.11216 | 1.000 | -0.1675 | 0.5645 |
| HBoV | HAdV | 0.54044^*^ | 0.07967 | 0.000 | 0.2805 | 0.8004 |
|  | FLUAV or FLUBV | 0.50242^*^ | 0.13905 | 0.014 | 0.0487 | 0.9562 |
|  | HPIV | 0.89222^*^ | 0.15400 | 0.000 | 0.3897 | 1.3947 |
|  | HMPV | 0.78488^*^ | 0.10450 | 0.000 | 0.4439 | 1.1259 |
|  | HRSV | 0.64448^*^ | 0.12029 | 0.000 | 0.2520 | 1.0370 |
|  | HRV | 0.40691^*^ | 0.07856 | 0.000 | 0.1505 | 0.6633 |
|  | Myco | 0.92688^*^ | 0.12711 | 0.000 | 0.5121 | 1.3417 |
|  | HAdV | 0.41804^*^ | 0.11003 | 0.007 | 0.0590 | 0.7771 |
|  | Coinfection† | 0.61652^*^ | 0.08890 | 0.000 | 0.3264 | 0.9066 |
| Coinfection† | No virus | -0.07608 | 0.08258 | 1.000 | -0.3456 | 0.1934 |
|  | HAdV | -0.11410 | 0.14074 | 1.000 | -0.5734 | 0.3452 |
|  | FLUAV or FLUBV | 0.27569 | 0.15553 | 1.000 | -0.2318 | 0.7832 |
|  | HPIV | 0.16836 | 0.10674 | 1.000 | -0.1800 | 0.5167 |
|  | HMPV | 0.02796 | 0.12224 | 1.000 | -0.3709 | 0.4268 |
|  | HRSV | -0.20961 | 0.08152 | 0.457 | -0.4756 | 0.0564 |
|  | HRV | 0.31036 | 0.12896 | 0.726 | -0.1105 | 0.7312 |
|  | Myco | -0.19849 | 0.11216 | 1.000 | -0.5645 | 0.1675 |
|  | HBoV | -0.61652^*^ | 0.08890 | 0.000 | -0.9066 | -0.3264 |

HAdV: Human adenovirus, FLUAV/FLUBV: Influenza virus A/B, HPIV: Human parainfluenza virus, HMPV: Human metapneumovirus, HRSV: Human respiratory syncytial virus, HRV: Human rhinovirus, Myco: Mycoplasma, HBoV: Human bocavirus.

†Coinfection describes patients who tested positive for more than two microorganisms.

*The mean difference is significant at the 0.05 level.

**Supplementary Table 7. (1)** **Comparison of CRP and microorganisms**

|  | | Patient  numbers | Mean | Standard  deviation | Standard error | 95% Confidence interval for mean | | Minimum | Maximum | Between- component variance |
| --- | --- | --- | --- | --- | --- | --- | --- | --- | --- | --- |
|  |  |  |  |  |  | Lower bound | Upper bound |  |  |  |
| No virus | | 806 | 2.1238 | 3.13703 | 0.11050 | 1.9069 | 2.3407 | 0.00 | 19.40 |  |
| HAdV | | 137 | 5.0717 | 4.09638 | 0.34998 | 4.3796 | 5.7638 | 0.10 | 21.80 |  |
| FLUAV or FLUBV | | 107 | 1.4570 | 2.60483 | 0.25182 | 0.9578 | 1.9563 | 0.00 | 15.80 |  |
| HPIV | | 297 | 1.6549 | 2.58672 | 0.15010 | 1.3595 | 1.9503 | 0.00 | 15.70 |  |
| HMPV | | 199 | 1.8638 | 2.88213 | 0.20431 | 1.4609 | 2.2667 | 0.00 | 18.20 |  |
| HRSV | | 868 | 1.0063 | 1.85676 | 0.06302 | 0.8826 | 1.1300 | 0.00 | 14.40 |  |
| HRV | | 172 | 2.0395 | 3.22832 | 0.24616 | 1.5536 | 2.5254 | 0.00 | 20.50 |  |
| Myco | | 248 | 2.2415 | 2.67305 | 0.16974 | 1.9072 | 2.5759 | 0.00 | 12.80 |  |
| HBoV | | 37 | 2.1754 | 2.56054 | 0.42095 | 1.3217 | 3.0291 | 0.10 | 9.80 |  |
| Coinfection† | | 510 | 2.2518 | 2.90090 | 0.12845 | 1.9995 | 2.5042 | 0.00 | 15.90 |  |
| Total | | 3381 | 1.9030 | 2.87064 | 0.04937 | 1.8062 | 1.9998 | 0.00 | 21.80 |  |
| Model | Fixed Effects |  |  | 2.75597 | 0.04740 | 1.8101 | 1.9959 |  |  |  |
|  | Random Effects |  |  |  | 0.36322 | 1.0813 | 2.7247 |  |  | 0.77466 |

CRP: C-reactive protein, HAdV: Human adenovirus, FLUAV/FLUBV: Influenza virus A/B, HPIV: Human parainfluenza virus, HMPV: Human metapneumovirus,

HRSV: Human respiratory syncytial virus, HRV: Human rhinovirus, Myco: Mycoplasma, HBoV: Human bocavirus.

†Coinfection describes patients who tested positive for more than two microorganisms.

**(2) Post-hoc analyses among groups by Bonferroni methods regarding CRP and microorganisms**

| Virus group 1 | Virus group 2 | Mean difference between groups  (Groups 1 and 2) | Standard error | Significance | 95% Confidence interval | |
| --- | --- | --- | --- | --- | --- | --- |
|  |  |  |  |  | Lower bound | Upper bound |
| No virus | HAdV | -2.94787^*^ | 0.25468 | 0.000 | -3.7791 | -2.1167 |
|  | FLUAV or FLUBV | 0.66680 | 0.28356 | 0.844 | -0.2586 | 1.5922 |
|  | HPIV | 0.46893 | 0.18708 | 0.551 | -0.1416 | 1.0795 |
|  | HMPV | 0.26004 | 0.21815 | 1.000 | -0.4519 | 0.9720 |
|  | HRSV | 1.11747^*^ | 0.13481 | 0.000 | 0.6775 | 1.5574 |
|  | HRV | 0.08427 | 0.23148 | 1.000 | -0.6712 | 0.8397 |
|  | Myco | -0.11772 | 0.20013 | 1.000 | -0.7708 | 0.5354 |
|  | HBoV | -0.05160 | 0.46336 | 1.000 | -1.5638 | 1.4606 |
|  | Coinfection† | -0.12803 | 0.15594 | 1.000 | -0.6369 | 0.3809 |
| HAdV | No virus | 2.94787^*^ | 0.25468 | 0.000 | 2.1167 | 3.7791 |
|  | FLUAV or FLUBV | 3.61467^*^ | 0.35556 | 0.000 | 2.4543 | 4.7751 |
|  | HPIV | 3.41680^*^ | 0.28463 | 0.000 | 2.4879 | 4.3457 |
|  | HMPV | 3.20791^*^ | 0.30596 | 0.000 | 2.2094 | 4.2064 |
|  | HRSV | 4.06534^*^ | 0.25336 | 0.000 | 3.2385 | 4.8922 |
|  | HRV | 3.03214^*^ | 0.31559 | 0.000 | 2.0022 | 4.0621 |
|  | Myco | 2.83015^*^ | 0.29337 | 0.000 | 1.8727 | 3.7876 |
|  | HBoV | 2.89627^*^ | 0.51061 | 0.000 | 1.2299 | 4.5627 |
|  | Coinfection† | 2.81984^*^ | 0.26521 | 0.000 | 1.9543 | 3.6854 |
| FLUAV or FLUBV | No virus | -0.66680 | 0.28356 | 0.844 | -1.5922 | 0.2586 |
|  | HAdV | -3.61467^*^ | 0.35556 | 0.000 | -4.7751 | -2.4543 |
|  | HPIV | -0.19787 | 0.31074 | 1.000 | -1.2120 | 0.8162 |
|  | HMPV | -0.40676 | 0.33038 | 1.000 | -1.4850 | 0.6715 |
|  | HRSV | 0.45067 | 0.28237 | 1.000 | -0.4709 | 1.3722 |
|  | HRV | -0.58253 | 0.33933 | 1.000 | -1.6900 | 0.5249 |
|  | Myco | -0.78452 | 0.31877 | 0.625 | -1.8248 | 0.2558 |
|  | HBoV | -0.71840 | 0.52561 | 1.000 | -2.4338 | 0.9970 |
|  | Coinfection† | -0.79483 | 0.29305 | 0.302 | -1.7512 | 0.1616 |
| HPIV | No virus | -0.46893 | 0.18708 | 0.551 | -1.0795 | 0.1416 |
|  | HAdV | -3.41680^*^ | 0.28463 | 0.000 | -4.3457 | -2.4879 |
|  | FLUAV or FLUBV | 0.19787 | 0.31074 | 1.000 | -0.8162 | 1.2120 |
|  | HMPV | -0.20889 | 0.25247 | 1.000 | -1.0328 | 0.6151 |
|  | HRSV | 0.064855^*^ | 0.18527 | 0.021 | 0.0439 | 1.2532 |
|  | HRV | -0.38465 | 0.26407 | 1.000 | -1.2465 | 0.4772 |
|  | Myco | -0.58665 | 0.23707 | 0.602 | -1.3603 | 0.1870 |
|  | HBoV | -0.52052 | 0.48047 | 1.000 | -2.0886 | 1.0475 |
|  | Coinfection† | -0.59696 | 0.20116 | 0.136 | -1.2535 | 0.0596 |
| HMPV | No virus | -0.26004 | .21815 | 1.000 | -0.9720 | 0.4519 |
|  | HAdV | -3.20791^*^ | .30596 | 0.000 | -4.2064 | -2.2094 |
|  | FLUAV or FLUBV | 0.40676 | .33038 | 1.000 | -0.6715 | 1.4850 |
|  | HPIV | 0.20889 | .25247 | 1.000 | -0.6151 | 1.0328 |
|  | HRSV | 0.85743^*^ | .21661 | 0.003 | 0.1505 | 1.5643 |
|  | HRV | -0.17577 | .28693 | 1.000 | -1.1122 | 0.7606 |
|  | Myco | -0.37776 | .26229 | 1.000 | -1.2338 | 0.4782 |
|  | HBoV | -.31164 | .49341 | 1.000 | -1.9219 | 1.2986 |
|  | Coinfection† | -.38807 | .23035 | 1.000 | -1.1398 | 0.3637 |
| HRSV | No virus | -1.11747^*^ | 0.13481 | 0.000 | -1.5574 | -0.6775 |
|  | HAdV | -4.06534^*^ | 0.25336 | 0.000 | -4.8922 | -3.2385 |
|  | FLUAV or FLUBV | -0.45067 | 0.28237 | 1.000 | -1.3722 | 0.4709 |
|  | HPIV | -0.64855^*^ | 0.18527 | 0.021 | -1.2532 | -0.0439 |
|  | HMPV | -0.85743^*^ | 0.21661 | 0.003 | -1.5643 | -0.1505 |
|  | HRV | -1.03320^*^ | 0.23002 | 0.000 | -1.7839 | -0.2825 |
|  | Myco | -1.23520^*^ | 0.19844 | 0.000 | -1.8828 | -0.5876 |
|  | HBoV | -1.16907 | 0.46264 | 0.520 | -2.6789 | 0.3408 |
|  | Coinfection† | -1.24551^*^ | 0.15376 | 0.000 | -1.7473 | -0.7437 |
| HRV | No virus | -0.08427 | 0.23148 | 1.000 | -0.8397 | 0.6712 |
|  | HAdV | -3.03214^*^ | 0.31559 | 0.000 | -4.0621 | -2.0022 |
|  | FLUAV or FLUBV | 0.58253 | 0.33933 | 1.000 | -0.5249 | 1.6900 |
|  | HPIV | 0.38465 | 0.26407 | 1.000 | -0.4772 | 1.2465 |
|  | HMPV | 0.17577 | 0.28693 | 1.000 | -0.7606 | 1.1122 |
|  | HRSV | 1.03320^*^ | 0.23002 | 0.000 | 0.2825 | 1.7839 |
|  | Myco | -0.20200 | 0.27347 | 1.000 | -1.0945 | 0.6905 |
|  | HBoV | -0.13587 | 0.49944 | 1.000 | -1.7658 | 1.4941 |
|  | Coinfection† | -0.21231 | 0.24301 | 1.000 | -1.0054 | 0.5808 |
| Myco | No virus | 0.11772 | 0.20013 | 1.000 | -0.5354 | 0.7708 |
|  | HAdV | -2.83015^*^ | 0.29337 | 0.000 | -3.7876 | -1.8727 |
|  | FLUAV or FLUBV | 0.78452 | 0.31877 | 0.625 | -0.2558 | 1.8248 |
|  | HPIV | 0.58665 | 0.23707 | 0.602 | -0.1870 | 1.3603 |
|  | HMPV | 0.37776 | 0.26229 | 1.000 | -0.4782 | 1.2338 |
|  | HRSV | 1.23520^*^ | 0.19844 | 0.000 | 0.5876 | 1.8828 |
|  | HRV | 0.20200 | 0.27347 | 1.000 | -0.6905 | 1.0945 |
|  | HBoV | 0.06613 | 0.48570 | 1.000 | -1.5190 | 1.6513 |
|  | Coinfection† | -0.01031 | 0.21335 | 1.000 | -0.7066 | 0.6860 |
| HBoV | HAdV | 0.05160 | 0.46336 | 1.000 | -1.4606 | 1.5638 |
|  | FLUAV or FLUBV | -2.89627^*^ | 0.51061 | 0.000 | -4.5627 | -1.2299 |
|  | HPIV | 0.71840 | 0.52561 | 1.000 | -0.9970 | 2.4338 |
|  | HMPV | 0.52052 | 0.48047 | 1.000 | -1.0475 | 2.0886 |
|  | HRSV | 0.31164 | 0.49341 | 1.000 | -1.2986 | 1.9219 |
|  | HRV | 1.16907 | 0.46264 | 0.520 | -0.3408 | 2.6789 |
|  | Myco | 0.13587 | 0.49944 | 1.000 | -1.4941 | 1.7658 |
|  | HAdV | -0.06613 | 0.48570 | 1.000 | -1.6513 | 1.5190 |
|  | Coinfection† | -0.07644 | 0.46923 | 1.000 | -1.6078 | 1.4549 |
| Coinfection† | No virus | 0.12803 | 0.15594 | 1.000 | -0.3809 | 0.6369 |
|  | HAdV | -2.81984^*^ | 0.26521 | 0.000 | -3.6854 | -1.9543 |
|  | FLUAV or FLUBV | 0.79483 | 0.29305 | 0.302 | -0.1616 | 1.7512 |
|  | HPIV | 0.59696 | 0.20116 | 0.136 | -0.0596 | 1.2535 |
|  | HMPV | 0.38807 | 0.23035 | 1.000 | -0.3637 | 1.1398 |
|  | HRSV | 1.24551^*^ | 0.15376 | 0.000 | 0.7437 | 1.7473 |
|  | HRV | 0.21231 | 0.24301 | 1.000 | -0.5808 | 1.0054 |
|  | Myco | 0.01031 | 0.21335 | 1.000 | -0.6860 | 0.7066 |
|  | HBoV | 0.07644 | 0.46923 | 1.000 | -1.4549 | 1.6078 |

CRP: C-reactive protein, HAdV: Human adenovirus, FLUAV/FLUBV: Influenza virus A/B, HPIV: Human parainfluenza virus, HMPV: Human metapneumovirus, HRSV: Human respiratory syncytial virus, HRV: Human rhinovirus, Myco: Mycoplasma, HBoV: Human bocavirus.

†Coinfection describes patients who tested positive for more than two microorganisms.

*The mean difference is significant at the 0.05 level.

**Supplementary Table 8. Correlations among key characteristics in the 3938 patients with respiratory infections**

|  | **Na** | **Age** | **Hosp** | **WBC** | **CRP** | **K** | **Cl** | **tCO2** | **Ca** | **Ps** | **AST** | **ALT** | **BUN** | **Cr** |
| --- | --- | --- | --- | --- | --- | --- | --- | --- | --- | --- | --- | --- | --- | --- |
| **Na** | . | (-)0.641 | (-)0.020 | (-)0.037 | (-)<0.0001 | 0.258 | <0.0001 | <0.0001 | <0.0001 | <0.0001 | (-)0.001 | (-)0.014 | (-)<0.0001 | <0.0001 |
| **Age** | (-)0.641 | . | 0.323 | (-)<0.0001 | <0.0001 | (-)<0.0001 | (-)<0.0001 | 0.994 | (-)<0.0001 | (-)<0.0001 | (-)<0.0001 | (-)<0.0001 | <0.0001 | <0.0001 |
| **Hosp** | (-)0.020 | 0.323 | . | 0.090 | <0.0001 | 0.123 | (-)0.037 | <0.0001 | (-)0.015 | (-)0.226 | <0.0001 | 0.008 | (-)<0.0001 | 0.340 |
| **WBC** | (-)0.037 | (-)<0.0001 | 0.090 | . | <0.0001 | <0.0001 | (-)0.086 | (-)<0.0001 | <0.0001 | 0.013 | (-)0.209 | 0.002 | 0.164 | (-)0.012 |
| **CRP** | (-)<0.0001 | <0.0001 | <0.0001 | <0.0001 | . | (-)<0.0001 | (-)<0.0001 | (-)<0.0001 | (-)<0.0001 | (-)<0.0001 | (-)<0.0001 | (-)0.010 | (-)<0.0001 | <0.0001 |
| **K** | 0.258 | (-)<0.0001 | 0.123 | <0.0001 | (-)<0.0001 | . | <0.0001 | <0.0001 | <0.0001 | <0.0001 | <0.0001 | <0.0001 | (-)<0.0001 | (-)<0.0001 |
| **Cl** | <0.0001 | (-)<0.0001 | (-)0.037 | (-)0.086 | (-)<0.0001 | <0.0001 | . | (-)<0.0001 | <0.0001 | <0.0001 | (-)0.024 | 0.844 | (-)<0.0001 | (-)<0.0001 |
| **tCO_2_** | <0.0001 | 0.994 | <0.0001 | (-)<0.0001 | (-)<0.0001 | <0.0001 | (-)<0.0001 | . | 0.006 | <0.0001 | (-)0.002 | (-)0.728 | (-)<0.0001 | (-)<0.0001 |
| **Ca** | <0.0001 | (-)<0.0001 | (-)0.015 | <0.0001 | (-)<0.0001 | <0.0001 | <0.0001 | 0.006 | . | <0.0001 | 0.001 | <0.0001 | (-)0.020 | (-)<0.0001 |
| **P** | <0.0001 | (-)<0.0001 | (-)0.226 | 0.013 | (-)<0.0001 | <0.0001 | <0.0001 | <0.0001 | <0.0001 | . | <0.0001 | <0.0001 | 0.100 | (-)<0.0001 |
| **AST** | (-)0.001 | (-)<0.0001 | <0.0001 | (-)0.209 | (-)<0.0001 | <0.0001 | (-)0.024 | (-)0.002 | 0.001 | <0.0001 | . | <0.0001 | 0.004 | (-)<0.0001 |
| **ALT** | (-)0.014 | (-)<0.0001 | 0.008 | 0.002 | (-)0.010 | <0.0001 | 0.844 | (-)0.728 | <0.0001 | <0.0001 | <0.0001 | . | 0.049 | (-)<0.0001 |
| **BUN** | (-)<0.0001 | <0.0001 | (-)<0.0001 | 0.164 | (-)<0.0001 | (-)<0.0001 | (-)<0.0001 | (-)<0.0001 | (-)0.020 | 0.100 | 0.004 | 0.049 | . | <0.0001 |
| **Cr** | <0.0001 | <0.0001 | 0.340 | (-)0.012 | <0.0001 | (-)<0.0001 | (-)<0.0001 | (-)<0.0001 | (-)<0.0001 | (-)<0.0001 | (-)<0.0001 | (-)<0.0001 | <0.0001 | . |

Na: Sodium, Hosp: Hospital stay (days), WBC: White blood cell, CRP: C-reactive protein, K: Potassium, Cl: Chloride, tCO_2_: Total carbon dioxide, Ca: Calcium, P: Phosphorus, AST: Aspartate aminotransferase, ALT: Alanine aminotransferase, BUN: Blood urea nitrogen, Cr: Creatinine.

**Supplementary Figures**

**Supplementary Figure 1. Distribution of sodium levels in 3938 patients with respiratory infections**


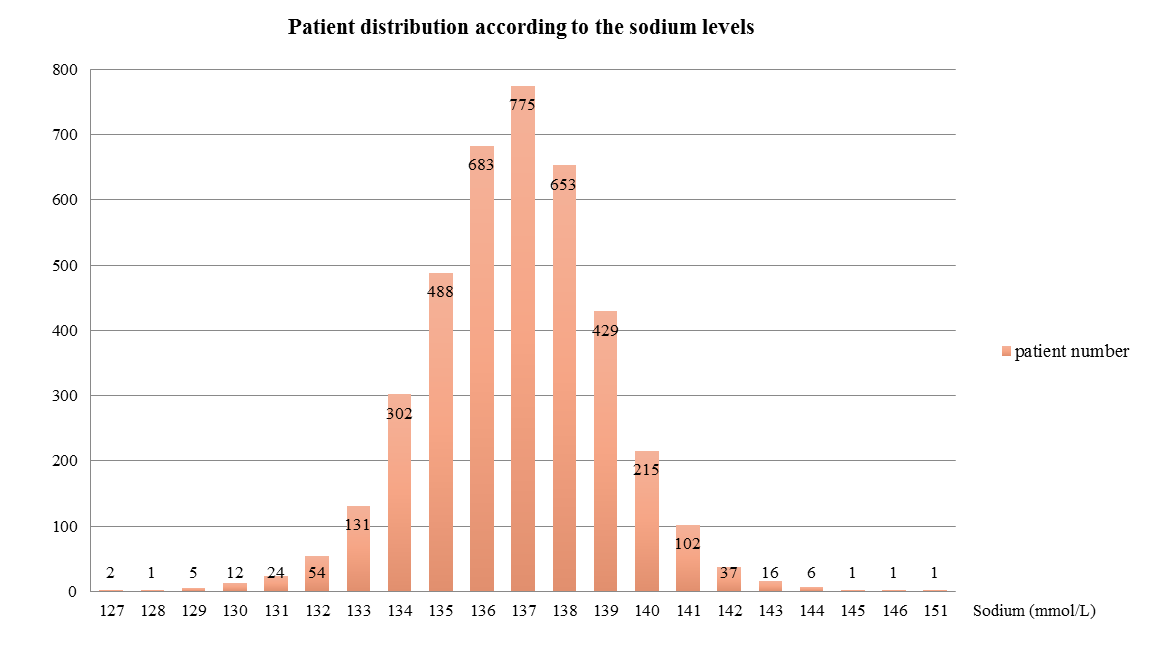


**Supplementary Figure 2. A receiver operating characteristic (ROC) curve of age (months), CRP, and BUN for the prediction of hyponatremia.** CRP: C-reactive protein, BUN: blood urea nitrogen


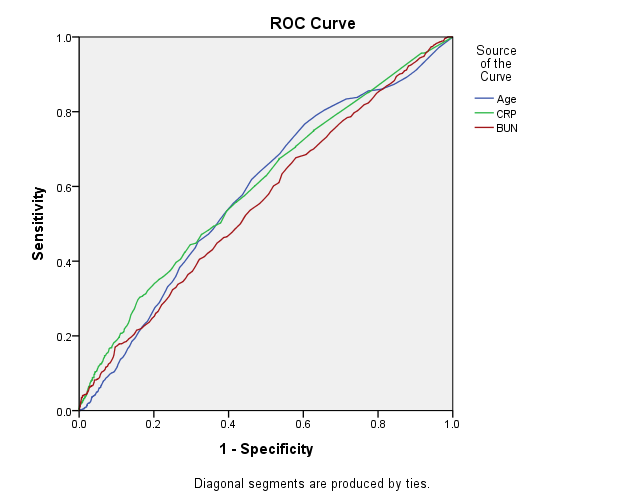

Supplement: Supplementary file 1 — Supplementary Dataset 10 [file 41598_2018_34703_MOESM1_ESM.doc]
